# Outcomes of hypothalamic oxytocin neuron-driven cardioprotection after acute myocardial infarction

**DOI:** 10.1007/s00395-023-01013-1

**Published:** 2023-10-06

**Authors:** Kathryn J. Schunke, Jeannette Rodriguez, Jhansi Dyavanapalli, John Schloen, Xin Wang, Joan Escobar, Grant Kowalik, Emily C. Cheung, Caitlin Ribeiro, Rebekah Russo, Bridget R. Alber, Olga Dergacheva, Sheena W. Chen, Alejandro E. Murillo-Berlioz, Kyongjune B. Lee, Gregory Trachiotis, Emilia Entcheva, Christine A. Brantner, David Mendelowitz, Matthew W. Kay

**Affiliations:** 1 Department of Biomedical Engineering, George Washington University, Suite 5000 Science and Engineering Hall, 800 22nd Street NW, Washington, DC 20052, USA; 2 Department of Pharmacology and Physiology, George Washington University, Suite 640 Ross Hall, 2300 Eye St. NW, Washington, DC 20052, USA; 3 Division of Cardiothoracic Surgery and Cardiothoracic Research, Veterans Affairs Medical Center, 50 Irving St. NW, Washington, DC 20422, USA; 4 The GWU Nanofabrication and Imaging Center, 800 22nd Street NW, Washington, DC 20052, USA; 5 Department of Anatomy, Biochemistry and Physiology, University of Hawaii, 651 Ilalo St, Honolulu, HI BSB 211 96813, USA

**Keywords:** Infarction, Cardioprotection, Parasympathetic nervous system, Oxytocin, Arrhythmia, Mitochondria

## Abstract

Altered autonomic balance is a hallmark of numerous cardiovascular diseases, including myocardial infarction (MI). Although device-based vagal stimulation is cardioprotective during chronic disease, a non-invasive approach to selectively stimulate the cardiac parasympathetic system immediately after an infarction does not exist and is desperately needed. Cardiac vagal neurons (CVNs) in the brainstem receive powerful excitation from a population of neurons in the paraventricular nucleus (PVN) of the hypothalamus that co-release oxytocin (OXT) and glutamate to excite CVNs. We tested if chemogenetic activation of PVN-OXT neurons following MI would be cardioprotective. The PVN of neonatal rats was transfected with vectors to selectively express DREADDs within OXT neurons. At 6 weeks of age, an MI was induced and DREADDs were activated with clozapine-N-oxide. Seven days following MI, patch-clamp electrophysiology confirmed the augmented excitatory neurotransmission from PVN-OXT neurons to downstream nuclei critical for parasympathetic activity with treatment (43.7 ± 10 vs 86.9 ± 9 pA; MI vs. treatment), resulting in stark improvements in survival (85% vs. 95%; MI vs. treatment), inflammation, fibrosis assessed by trichrome blue staining, mitochondrial function assessed by Seahorse assays, and reduced incidence of arrhythmias (50% vs. 10% cumulative incidence of ventricular fibrillation; MI vs. treatment). Myocardial transcriptomic analysis provided molecular insight into potential cardioprotective mechanisms, which revealed the preservation of beneficial signaling pathways, including muscarinic receptor activation, in treated animals. These comprehensive results demonstrate that the PVN-OXT network could be a promising therapeutic target to quickly activate beneficial parasympathetic-mediated cellular pathways within the heart during the early stages of infarction.

## Introduction

Disruption of cardiac parasympathetic tone is a hallmark of cardiovascular disease, including myocardial infarction (MI) [115]. Marked autonomic imbalance with reduced parasympathetic cardiac vagal activity [54] that precedes sympathetic overactivity is observed early after the onset of disease, as described for heart failure [46, 83]. Reduced vagal drive to the heart is also a strong independent risk factor for life-threatening arrhythmias and sudden cardiac death post-MI [21, 37, 98]. Targeted elevations of cardiac vagal activity using implanted devices is cardioprotective during and after acute MI and heart failure [14, 80, 115]. Increased vagal activity improves survival and cardiac function following MI in small and large animal models, suggesting that activation of the cholinergic pathway is therapeutic [2, 63, 65, 75, 114, 115]. However, a rapid non-invasive approach to increase parasympathetic activity in patients with unanticipated acute onset cardiovascular events, such as MI, is lacking.

The paraventricular nucleus of the hypothalamus (PVN) is an important site of autonomic control, and there are direct excitatory projections from PVN oxytocin (OXT) neurons to parasympathetic cardiac vagal neurons (CVNs) in the brainstem. Targeted elevation of hypothalamic PVN-OXT neuron activity increases the downstream activity of brainstem CVNs that control parasympathetic drive to the heart [34]. In an animal model of heart failure, chronic chemogenetic elevation of PVN-OXT neuron activity improved left ventricular (LV) electrophysiology and contractile function, reduced hypertrophy and fibrosis, and improved LV sensitivity to β-adrenergic stimulation [29, 34, 131]. In an animal model of obstructive sleep apnea, chronic chemogenetic activation of PVN-OXT neurons increased CVN activity and prevented the hypertension that occurred in untreated animals [49]. In animals that were exposed to chronic intermittent hypoxia (CIH) and developed hypertension, chronic chemogenetic activation of PVN-OXT neurons blunted the progression of hypertension and conferred cardioprotection during an additional 4 weeks of CIH exposure [97]. In obstructive sleep apnea patients, intranasal OXT increased indices of parasympathetic activity, including heart rate variability and total sleep time [47, 48]. These results highlight PVN-OXT neurons as a source of therapeutic parasympathetic drive, yet it is unknown whether this treatment paradigm provides benefits during acute MI and what cellular and molecular changes occur in the heart following PVN-OXT neuron activation that would preserve cardiac function after MI.

To study the effects of PVN-OXT neuron activation following MI, a chemogenetic approach with selective expression of Designer Receptors Exclusively Activated by Designer Drugs (DREADDs) was used to chronically activate PVN-OXT neurons in rats [49]. In 1-week old pups, DREADDs and channelrhodopsin (ChR2) expression was targeted to PVN-OXT neurons using three viral vectors and Cre-Lox recombination [29, 49, 131]. At 3 weeks of age, a telemetry device to measure the ECG was implanted subcutaneously in all rats to measure heart rate and detect arrhythmias. At 6 weeks of age, an MI was induced by permanent ligation of the LAD coronary artery. Seven days after MI or sham MI surgery, *in vivo* and *ex vivo* cardioprotective outcomes were assessed.

## Materials and methods

### Experimental design

**Fig. 1:**
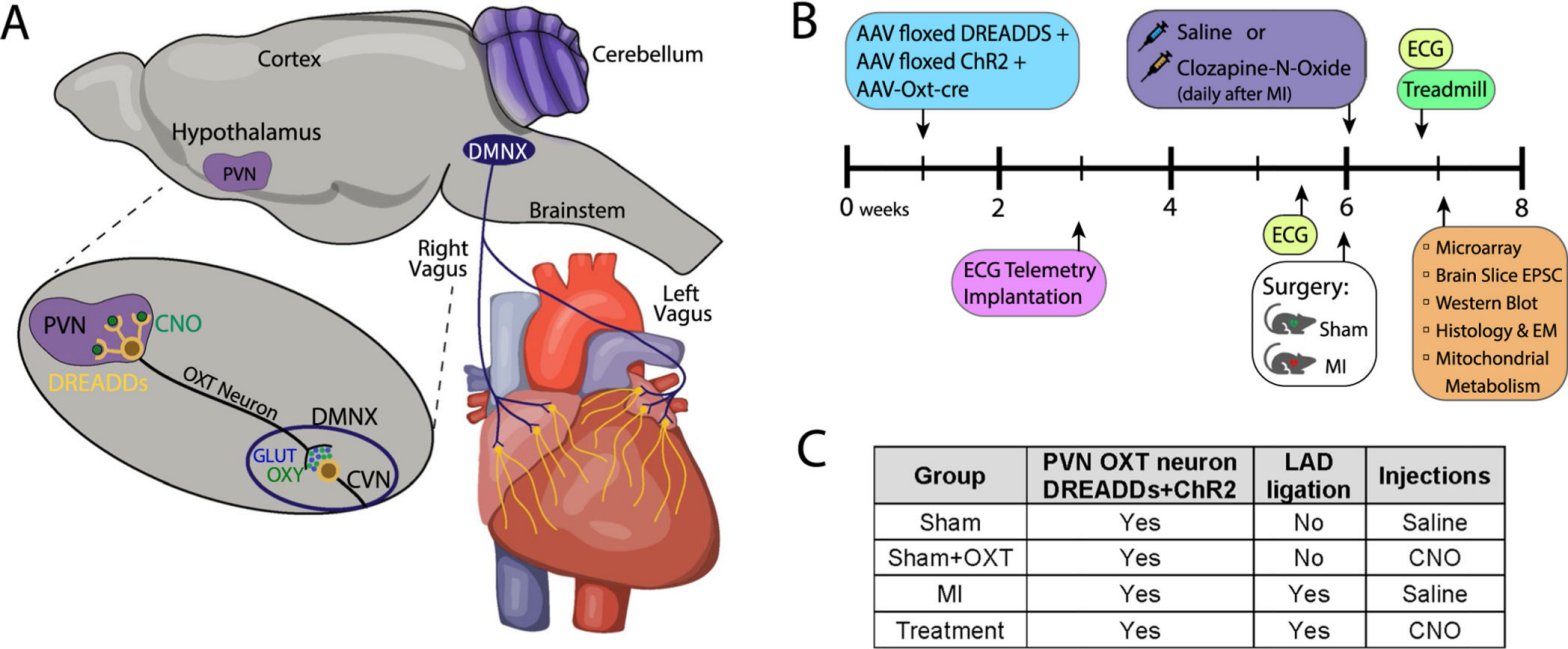
**A** In vivo activation of brainstem parasympathetic neurons. Selective expression of excitatory hM3D(Gq) DREADDs within PVN-OXT neurons, and subsequent activation via CNO, increases the firing rate of PVN-OXT neurons, which co-release OXT and GLUT at synapses on parasympathetic DMNX neurons of the medulla. Elevated release of synaptic OXT and GLUT increases excitatory neurotransmission to DMNX parasympathetic neurons, elevating their firing rate to increase downstream activation of post-ganglionic parasympathetic ganglia neurons that release acetylcholine at their target tissue. Ultimately, the increased release of acetylcholine within the myocardium elevates the level of myocyte muscarinic pathway activation via cholinergic muscarinic (Chrm2/M2) receptors. **B** Protocol timeline from animal birth to sacrifice at 7 weeks of age with subsequent *ex vivo* assessments. Three viruses encoding DREADDs, ChR2, and OXT-Cre were injected into the PVN of all rats at 1 week of age, followed by surgical implantation of an ECG transmitter at 3 weeks of age. Baseline ECG and HRR data were collected at 5 weeks, followed by either sham MI or MI surgery. Immediately following surgery, and daily for 7 days, animals were injected with either saline or CNO. At 7 weeks of age (or 1-week post-MI), animals were sacrificed, and brains and hearts were collected for *ex vivo* assessments. **C** The four animal groups with the assigned interventions are shown in the table. Abbreviations: PVN, paraventricular nucleus of the hypothalamus; DMNX, dorsal motor nucleus of the vagus; DREADDs, designer receptor exclusively activated by designer drugs; CNO, clozapine-N-oxide; OXT, oxytocin; GLUT, glutamate; CVN, cardiac vagal neuron; AAV, adeno-associated virus; ChR2, channelrhodopsin; EPSC, excitatory post-synaptic current; EM, electron microscopy

PVN-OXT neurons were chronically activated *in vivo* using Cre-Lox recombination and an OXT promoter [49]. Axons projecting from those PVN-OXT neurons synapse with CVNs that reside within the dorsal motor nucleus of the vagus (DMNX) of the brainstem (Fig. 1A). Our prior work has demonstrated that DREADDs-mediated activation of PVN-OXT neurons causes acute reductions in heart rate and blood pressure that are the result of increased cardiac vagal tone [34, 49]. This approach, combined with a surgically induced LV MI, was implemented in male Sprague–Dawley rats in an experimental protocol beginning at 1 week of age and ending at 7 weeks of age (Fig. 1B). All animal procedures were completed in agreement with the George Washington University institutional guidelines and in compliance with the panel of Euthanasia of the American Veterinary Medical Association and the National Institutes of Health (NIH) *Guide for the Care and Use of Laboratory Animals*.

### DREADDs and ChR2 expression

Three viral vectors and Cre-Lox recombination were used for selective and robust expression of DREADDs and channelrhodopsin (ChR2) within PVN-OXT neurons (Fig. 1A) [29, 49, 131]. Expression of the enzyme Cre recombinase was exclusively driven by the OXT promoter of an adenovirus (AAV1-OXT-Cre) that was co-injected with vectors for floxed ChR2 (AAV1-EF1a-DIO-hChR2, H134R) and floxed excitatory DREADDs (AAV2-hSyn-DIO-hM_3_D(Gq)-mCherry) [49]. Previous work showed, using immunohistochemical analysis, that this viral system elicits high selectivity (83.1 ± 2.1% and 93 ± 2.0%) for the expression of DREADDs and ChR2, respectively, in PVN-OXT neurons [49, 91]. At 1 week of age, all animals were anesthetized by hypothermia and mounted in a stereotactic apparatus (Stoelting). The skull was opened and a calibrated pipette containing the adenoviral cocktail was directed into the PVN and 30–50 nL of viral mixture was microinjected over 20 min. The pipette was left in place for 5 min prior to careful and slow retraction. Within 2 weeks, PVN-OXT neurons stably expressed DREADDs, and activation of DREADDs in PVN-OXT neurons by the ligand clozapine-N-Oxide (CNO, 1 mg/Kg) increased neuronal firing and reduced heart rate and blood pressure [34, 49].

### Experimental groups

One week after administering the adenoviral cocktail to the PVN, each animal was randomly assigned to one of four groups: **Sham** (sham MI and saline injections), **Sham + OXT** (sham MI and CNO injections), **MI** (MI and saline injections), and **Treatment** (MI and CNO injections). Investigators were double blinded. DREADDs expressed in PVN-OXT neurons were activated in Sham + OXT and Treatment animals via intraperitoneal injections of CNO (1 mg/Kg, 200–250 μL), the DREADDs ligand. Sham and MI animals received saline-only intraperitoneal injections (200–250 μL) that did not activate DREADDs. To determine if CNO had any off-target effects, we studied two supplemental groups of MI animals that did not have DREADDs expression. One group received saline (*n* = 8), the other group received CNO (*n* = 8). Results from these two untreated MI groups that did not express DREADDs did not differ from the untreated MI group that did express DREADDs, indicating that any off-target effects of CNO were insignificant. Results from MI animals that did not have DREADDs expression are presented in Supplemental Fig 5.

The initial CNO injection for all animals was given within 15 min after sham or LAD artery ligation. One CNO injection was given daily thereafter for 7 days, after which animals were sacrificed for *ex vivo* assessments and tissue analysis (Fig. 1B). Animal numbers for each experimental group (excluding the two supplemental groups) for each assessment are listed in Table 1.

### Telemetry device implantation and induction of MI

At 3 weeks of age, a device (DSI ETA-F10) to measure the ECG was implanted subcutaneously (Lead II configuration) in all rats to measure heart rate, detect arrhythmias, and assess QRST morphology (Fig. 1B). At 6 weeks of age, a thoracotomy was performed in all rats to induce MI by permanent ligation of the LAD coronary artery. A 6–0 silk suture was passed around the left coronary artery two thirds of the way between its origin near the pulmonary conus and the cardiac apex. The suture was tied to ligate the artery in all MI and Treatment rats. LAD occlusion was confirmed upon observing a well-defined area of epicardial cyanosis with regional hypokinesia and ECG changes. In all Sham and Sham + OXT rats, the suture was not tied and was removed (sham MI).

### *In vivo* ECG assessments

#### ECG and arrhythmia analysis

The ECG of each animal was recorded continuously for 24 h before and immediately after the MI or sham MI surgery (Fig. 1B). Five days after each surgery, the ECG was continuously recorded again for 24 h. All ECG recordings were then analyzed using LabChart software (AD Instruments) to measure standard QRST parameters, including ST segment height and QRS duration. The incidence of ventricular tachycardia (VT) and ventricular fibrillation (VF) was also measured for the 24-h interval immediately after each MI or sham MI surgery.

#### Heart rate recovery after peak running effort

Five days after MI surgery (Fig. 1B), rapid reductions in HR after animals reached their peak effort of treadmill running were analyzed to assess vagal activity [21]. Animals began with an initial warm up period of 5 min at a treadmill speed of 6 cm/sec. Speed was then increased to 12 cm/sec and increased by 6 cm/sec every 3 min until peak running effort was reached: the moment when animals would no longer run; at which time the treadmill was stopped. Heart rate recovery (HRR) was quantified by calculating the time required for HR to recover to 95%, 90%, and 85% of the HR that occurred during peak running effort.

### *Ex vivo* function assessments

Seven days after MI or sham MI surgery, animals were sacrificed to either conduct (1) patch-clamp electrophysiology studies of brainstem CVNs or (2) flow cytometric and respiration assays of mitochondria isolated from LV myocardium.

#### Brainstem excitatory post-synaptic currents evoked by photostimulation of PVN-OXT fibers that express ChR2

Neurotransmission from PVN-OXT neurons to CVNs is a major source of CVN excitatory input [24, 91, 92]. To measure changes in this neurotransmission, stimulation of PVN synaptic terminals that monosynaptically synapse upon DMNX neurons was achieved by selective expression of ChR2 in PVN-OXT neurons and their fibers surrounding DMNX neurons using a floxed ChR2 (AAV1-EF1a-DIO-hChR2, H134R) vector, as described above and in our previous studies [24, 92]. Excitatory post-synaptic currents (EPSCs) evoked by photostimulation of ChR2-expressing PVN-OXT fibers were examined in ex vivo brainstem slices to determine if the MI, and if daily activation of PVN-OXT neurons in Treatment animals, altered this excitatory neurotransmission.

To obtain viable brain slices, animals were transcardially perfused with ice-cold glycerol-based artificial cerebrospinal fluid containing (mmol/L): 252 glycerol, 1.6 KCL, 1.2 NaH2PO4, 1.2 MgCl, 2.4 CaCl2, 26 NaHCO3, and 11 glucose. The brain was carefully removed and brainstem slices of 300 μm thickness containing brainstem vagal neurons were obtained using a vibratome. Brain slices were transferred to a solution comprising (mmol/L): 110 N-methyl D-glucamine, 2.5 KCl, 1.2 NaH2PO4, 25 NaHCO3, 25 glucose, 110 HCl, 0.5 CaCl2, and 10 MgSO4 equilibrated with 95% O2–5% CO2 (pH 7.4) at 37 °C for 15 min. Slices were then transferred to a superfusion recording chamber containing (mmol/L) 125 NaCl, 3 KCl, 2 CaCl2, 26 NaHCO3, 5 glucose, and 5 HEPES equilibrated with 95 O2–5% CO2 (pH 7.4) at 25 °C. Each slice was equilibrated in this solution for at least 30 min before experiments commenced. Neurons in the dorsal motor nucleus of the vagus (DMNX), where para-sympathetic CVNs are localized, were visualized using differential interference contrast optics. Patch pipettes (2.5–3.5 MΩ) were filled with a solution consisting of (mmol/L) 135 K-gluconic acid, 10 HEPES, 10 ethylene glucol-bis (β-aminoethyl ether)-N,N,N′N′-tetraacetic acid, 1 CaCl2, and 1 MgCl2, and pH 7.35. Cell bodies in the DMNX were then patched and voltage clamp whole-cell recordings were made at a holding potential of − 80 mV with an Axopatch 200 B and pClamp 8 software (Axon Instruments). ChR2-expressing PVN-OXT fibers were photostimulated using a blue laser (473 nm, CrystaLaser, Reno, NV, USA). Laser light pulses were applied for 3 ms duration at 1 Hz, intensity was maintained across all experiments at 10 mW. To confirm that the EPSCs were glutamatergic, at the end of each experiment, d-2-amino-5-phosphonovalerate (APV, 50 mM) and 6-cyano-7-nitroquinoxaline-2,3-dione (CNQX,50 mM) were applied to block glutamatergic NMDA and AMPA/kainate receptors, respectively. Differences in post-synaptic transmission from PVN-OXT fibers between groups were assessed by comparing the amplitudes of the photoactivated EPSCs.

#### LV mitochondria isolation

In a separate set of animals, hearts were rapidly excised, the aorta was cannulated, and the coronary arteries were flushed of blood with ice-cold modified Krebs–Henseleit solution (mmol/L): 118 NaCl, 4.7 KCl, 1.25 CaCl_2_, 0.57 MgSO_4_, 1.17 KH_2_PO_4_, 25 NaHCO_3_, and 6 glucose. The LV free wall distal to the suture was removed and processed for mitochondrial isolation as described by Makinen and Lee [76]. Briefly, tissue was weighed and placed in nine volumes of ice-cold isolation buffer (IB, in mmol/L): 280 sucrose, 10 HEPES, 1 EDTA, 1 EGTA, and pH 7.2. Protease (Subtilisin A; Sigma-Aldrich) was added (5 mg per g wet muscle), and continually minced and mixed for 7 min. An equal volume of solution IB was added to end digestion. The mince was homogenized with an T25 digital ULTRA-TURRAX (IKA Works, Inc.) homogenizer for 30 s at 6000 rpm. The homogenate was then centrifuged at 700x**g** for 10 min in a refrigerated (4 °C) centrifuge (Beckman J2–21 M/E) to pellet down contractile protein and cellular debris. The supernatant was rapidly decanted through a double layer of cheesecloth and centrifuged at 10,000x**g** for 10 min to pellet down the mitochondrial fraction. The supernatant was discarded, and the mitochondrial pellet was resuspended and washed in a volume equal to the original homogenate in solution IB, and centrifuged at 7500x**g** for 10 min. The supernatant was discarded, and the final mitochondrial pellet was suspended in 500 μl of mitochondrial assay solution (MAS, mmol/L: 220 mannitol, 70 sucrose, 10 KH2PO4, 5 MgCl2, 2 HEPES, and pH 7.4) for a yield of 19–24 mg/ml of mitochondrial protein.

#### Isolated mitochondria assays

Flow cytometric analysis of isolated mitochondria was performed to measure mitochondrial size, complexity, and superoxide production after labeling with 200 nM MitoTracker Green (Invitrogen) and 2.5 μmol/L MitoSOX red (Invitrogen) for 15 min at 37 °C. Mitochondrial size (forward scatter, FSC), complexity (side scatter, SSC), or superoxide production (MitoSOX red fluorescence intensity) were measured using a Cytek Aurora Flow Cytometer. Data are shown as histograms and as bar graphs of average signal intensity for ~ 120,000 ungated events.

Extracellular flux (XF) assays were performed using an Agilent Seahorse XFe96 Analyzer. Isolated mitochondria were diluted in MAS with 0.2% (w/v) bovine serum albumin (Sigma-Aldrich A-7030; fatty acid content < 0.01%), for a final protein content of 4 ug per assay well. Altered mitochondrial respiration was assessed using tandem coupling and electron flow assays. The coupling assay measured function/dysfunction between the electron transport chain (ETC) and the oxidative phosphorylation (OXPHOS) machinery. The electron flow assay measured sequential electron flow through the complexes of the electron transport chain to identify sites of mitochondrial dysfunction or modulation.

##### Coupling assay:

Isolated mitochondria were loaded into wells in a coupled state (State II) with 10 mmol/L succinate and 2 μmol/L rotenone as substrate, then centrifuged at 2000x**g** for 20 min at 4 °C. Drug port injections were as follows: State III was initiated with ADP (4 mmol/L final), State IV induced with the addition of oligomycin (2.5 μg/ml final) (State IVo), and carbonyl cyanide-4 (trifluoromethoxy) phenylhydrazone (FCCP)-induced maximal uncoupled respiration (4 μmol/L) (State IIIu). Each state was sequentially measured, allowing oxygen consumption rates to be assessed as previously described by others [15, 30].

##### Electron flow assay:

Isolated mitochondria were loaded into wells in an uncoupled state with 10 mmol/L pyruvate, 2 mmol/L malate, and 4 μmol/L FCCP as substrate, then centrifuged at 2000x**g** for 20 min at 4 °C. Drug port injections were as follows: 2 μmol/L final rotenone, 10 mmol/L final succinate, 4 μmol/L final antimycin A, 10 mmol/L ascorbate, and 100 μmol/L N1, N1, N1, N1-tetramethyl-1,4-phenylene diamine (TMPD). Oxygen consumption rate was measured after each port injection.

### Western blots and transcriptomics

Seven days after MI or sham MI surgery, a separate set of animals was sacrificed to conduct western blots and transcriptome profiling. Hearts were rapidly excised and rinsed of blood, as described above, and then flash frozen in liquid nitrogen. The ischemic (**I**) zone and the healthy or remote (**R**) zone were sectioned. The I zone consisted of the LV free wall located mid- to apically distal to the LAD ligature, including peri-infarct, core infarct, and minimal healthy tissue. The R zone consisted of the base of the septum and right ventricle. These sections were then cryo-pulverized.

#### Protein extraction and western blotting

Cryo-pulverized tissue from the I and R zones was lysed in RIPA buffer with protease inhibitors (Roche) then sonicated and spun at 12,000*x***g** at 4° for 15 min. 10–30 ug of total protein was loaded into 4–15% Criterion TGX precast BioRad gels and ran in reducing conditions. Protein was transferred onto BioRad PVDF membranes, blocked in 3% non-fat milk or 3% BSA at room temperature for 2 h, then incubated overnight at 4 °C with primary antibodies for Serca2a (mouse monoclonal, 1:1000; Santa Cruz), IL-1β (mouse monoclonal, 1:1000; Cell Signaling Technologies) or GAPDH (rabbit polyclonal,1:7000; Sigma). Membranes were then washed and incubated with an HRP conjugated anti-mouse or anti-rabbit secondary antibody (BioRad) for 2 h at room temperature. Chemiluminescent blots were developed with Radiance ECL on an Azure c600 Western blot imaging system.

Because of the large number of animals in each group, not all samples could be loaded on the same gel. Two gels were, therefore, run that included samples from each of the four groups on each gel, having specific samples repeated on each gel. Signal intensity was then normalized between gels using the blot intensities of the repeated samples, as in our previous studies [29]. This method provides consistent normalization of all samples both within each group and across the minimum number of required gels.

### RNA extraction and transcriptome profiling

Total RNA was isolated from cryo-pulverized tissue from the I zone using a Qiagen RNeasy Fibrous Tissue Mini Kit according to the manufacturer’s protocol. Extracted RNA was quantified spectrophotometrically with NanoDrop One/Onec (Thermo Fisher Scientific), and RNA quality was assessed using an Agilent 2100 Bioanalyzer. Transcriptome profiling was performed using the Affymetrix Clariom S GeneChip according to the manufacturer’s instructions. Raw data were analyzed using Affymetrix Expression Console and Transcriptome Analysis Console software prior to downstream analysis. Statistically significant differentially expressed genes (DEGs) were identified using an expression fold change (FC) > 1.7 or < − 1.7 and a false discovery rate of *p* < 0.08. Semi-quantitative real-time polymerase chain reaction (PCR) of select DEGs was performed to confirm the expression fold changes measured using the Affymetrix Clariom S GeneChip (Figs. S2 and S3).

### Histological assessments

Seven days after MI or sham MI surgery, a separate set of animals was sacrificed to analyze myocardial tissue structure and mitochondrial ultrastructure within the ischemic zone. Hearts were rapidly excised and rinsed of blood, as described above.

#### Tissue histology

Excised hearts were fixed in 4% paraformaldehyde, then embedded in paraffin, and 7 μm thick transverse sections were cut beginning distal to the LAD ligature and moving toward the apex. Sections containing the core of the ischemic zone were mounted on glass slides, stained with Masson’s trichrome stain to highlight collagen deposition, and scanned using a Panoramic III scanner (Epredia and 3D Histech) at 20x. Digital images were analyzed using QuantCenter software (3D Histech) to calculate the ratio of collagen fiber area to total tissue area per heart section.

#### Electron microscopy

Cubes of tissue (2–3 mm^3^) were harvested from the medial LV free wall of the I zone of each heart and processed for transmission electron microscopy (TEM), generally following our previously described approach [51]. Immediately after harvest, each tissue cube was placed in fixative solution (2.5% glutaraldehyde and 1% paraformaldehyde in 0.1 M sodium cacodylate buffer). Cubes were then post-fixed for 1 h in 1% osmium tetroxide solution and stained overnight with a 1% uranyl acetate solution. The following day, cubes were dehydrated in a series of ethanol washes and embedded in Embed812 resin blocks. The blocks were ultrathin sectioned (95 nm thickness) and sections were stained with uranyl acetate and lead citrate to enhance contrast. Transmission electron micrographs were recorded using an FEI Talos F200X electron microscope at 4300 × magnification. Ten images per sample were randomly chosen for analysis, totaling approximately 823 μm^2^ of tissue and roughly 388 mitochondria per heart. ImageJ (NIH) was used to quantify mitochondrial aspect ratio (major axis/minor axis), size, and quantity.

### Statistics

Animal numbers for each experimental outcome are provided in each figure caption and listed in Table 1. All data are presented as mean ± standard deviation (SD) and analyzed using GraphPad Prism. Comparisons between groups were performed by one- or two-way ANOVA followed by Tukey’s multiple comparisons test, unless noted otherwise. For survival analysis, Kaplan–Meier curves were plotted and compared using the log-rank test. For ECG analyses, QRST indices and incidence of VT and VF were compared using one-way ANOVA with Tukey’s multiple comparisons test and two-way ANOVA with Sidak’s multiple comparisons test, respectively. Significance was accepted at *p* < 0.05 and denoted as * for *p* < 0.05, ** for *p* < 0.01, *** for *p* < 0.001, **** for *p* < 0.0001.

## Results

### Excitatory neurotransmission

**Fig. 2:**
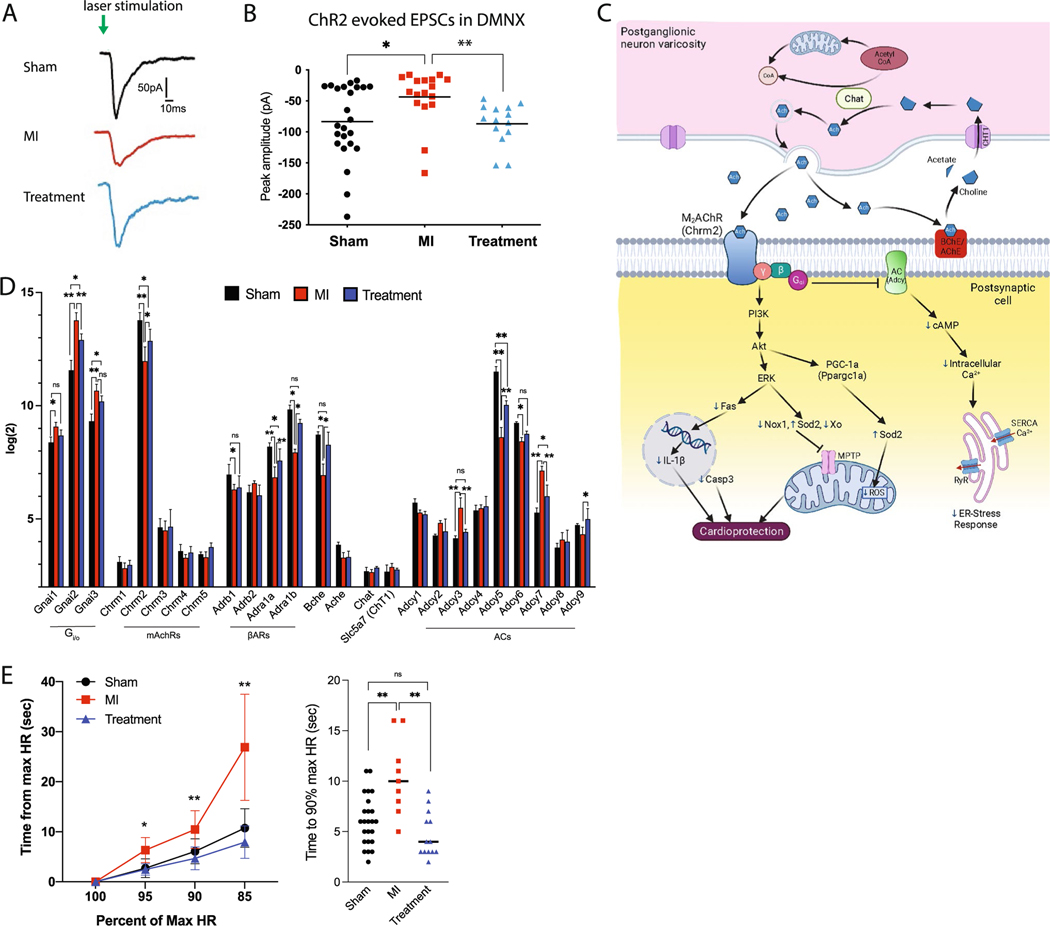
Daily activation of PVN-OXT neurons after MI maintained excitatory neurotransmission to parasympathetic neurons in the DMNX and rapid HRR. **A** Typical voltage clamp recordings of evoked EPSCs upon photoexcitation of ChR2-expressing PVN-OXT neurons show diminished EPSC amplitude for MI animals. **B** Distribution of evoked EPSC amplitudes recorded 7 days after MI for each group illustrates maintenance of EPSC in Treatment and loss of EPSC in MI animals. EPSC amplitude was significantly lower in MI animals (− 44 ± 10 pA) compared to Sham (− 84 ± 12 pA) and Treatment animals (− 87 ± 9 pA) (Kruskal–Wallis test with post hoc Dunn’s test, mean ± SEM, *p* = 0.0045; *p = 0.02 and **p = 0.007). Sham: *n* = 24 cells, 24 brainstem slices, 11 animals; MI: *n* = 17 cells, 17 brainstem slices, 6 animals; Treatment: *n* = 14 cells, 14 brainstem slices, 5 animals. **(C)** A cholinergic synapse showing ACh production and release and the pathways that are activated by the primary Gi/o coupling of the muscarinic ACh type 2 receptors (m_2_AChRs) of the post-synaptic cell. Preserved gene expression of myocyte m_2_AChRs and elevated release of ACh from cardiac cholinergic axon varicosities could activate cellular cardioprotective pathways that would reduce sarcoplasmic reticulum (SR) stress and mitochondrial ROS, inhibit activation of the mitochondrial permeability transition pore (MPTP), and reduce nuclear production of inflammatory cytokines. Small blue arrows indicate increased or decreased abundance/activity. **D** Gene expression profiles of proteins that are integral for myocyte muscarinic signaling (*n* = 3 per group; student’s *t* test; mean ± SD; *p < 0.05). **E** HRR time 5 days after MI as a percentage of HR at peak running effort (the maximum HR). Recovery time to 95%, 90%, and 85% of maximum HR was significantly longer for MI animals. HRR time was not significantly different between Treatment and Sham animals (Sham *n* = 24; MI, *n* = 9; Treatment, *n* = 13; two-way ANOVA; mean ± SD, *p < 0.05, **p < 0.01). Abbreviations: PVN, paraventricular nucleus; OXT, oxytocin; CVN, cardiac vagal neuron; ChR2, channelrhodopsin; EPSC, excitatory post-synaptic current; HR, heart rate; HRR, heart rate recovery

Seven days after MI or sham MI surgery, the amplitude of excitatory post-synaptic currents (EPSCs) in DMNX neurons upon photoexcitation of hypothalamic ChR2-expressing PVN-OXT fibers (Fig. 1A) was 83.5 ± 12pA (mean ± SEM) in Sham animals (Fig. 2A, B). EPSC amplitude was significantly reduced in MI animals (43.7 ± 10 pA) yet there was no significant difference between Treatment (86.9 ± 9 pA) and Sham animals. We observed robust ChR2 expression in fibers from PVN–OXT neurons in the DMNX in each of the 22 animals used for these experiments. These results indicate that excitatory neurotransmission from hypothalamic PVN-OXT neurons to parasympathetic DMNX neurons was diminished in MI animals and was maintained in both Sham and Treatment animals.

Maintenance of excitatory parasympathetic activity to cholinergic receptors within the heart is vital for the cardioprotective outcomes of restoring autonomic balance. Gene expression levels for key components of the cholinergic synapse (Fig. 2C, D) indicated significant loss of muscarinic acetylcholine (ACh) receptor M2 (mAchR2/Chrm2) following MI and indicated its preservation in Treatment animals. Semi-quantitative real-time PCR confirmed this outcome (Fig S2). There was no change in expression of G-protein coupled receptor β or ϒ subunits; however, Gi/o subunits (Gnai1–3) were upregulated in MI with a trend toward their preservation in Treatment animals (Fig. 2D). Adrenergic α and β receptor (Adra1a-b, Adrb1) expression was downregulated in MI and preserved in Treatment animals. There were no changes in the gene expression of ACh synthesis or transport enzymes; however, butyrylcholinesterase (Bche), a homolog of acetylcholinesterase (Ache) was significantly reduced in MI animals and preserved in Treatment animals. The gene expression of several adenylyl cyclase isoforms was dysregulated in MI and Treatment animals, suggesting a reduction in cAMP synthesis, and a blunted sympathetic response (Fig. 2D).

One functional benefit of potentially maintained muscarinic pathway activation in Treatment animals was the maintenance of rapid HRR after peak running effort, consistent with the correlation between Chrm2 gene polymorphism and HRR in humans [38]. The HRR of MI animals was significantly slower than Sham animals at each measured percentage of the maximum heart rate during peak effort while the HRR of Treatment animals was no different than Sham (Fig. 2E). These results suggest that parasympathetic transmission from hypothalamic PVN-OXT fibers to cardiac cholinergic synapses was similar for Sham and Treatment animals and was impaired for MI animals.

### Transcriptome profiling

**Fig. 3:**
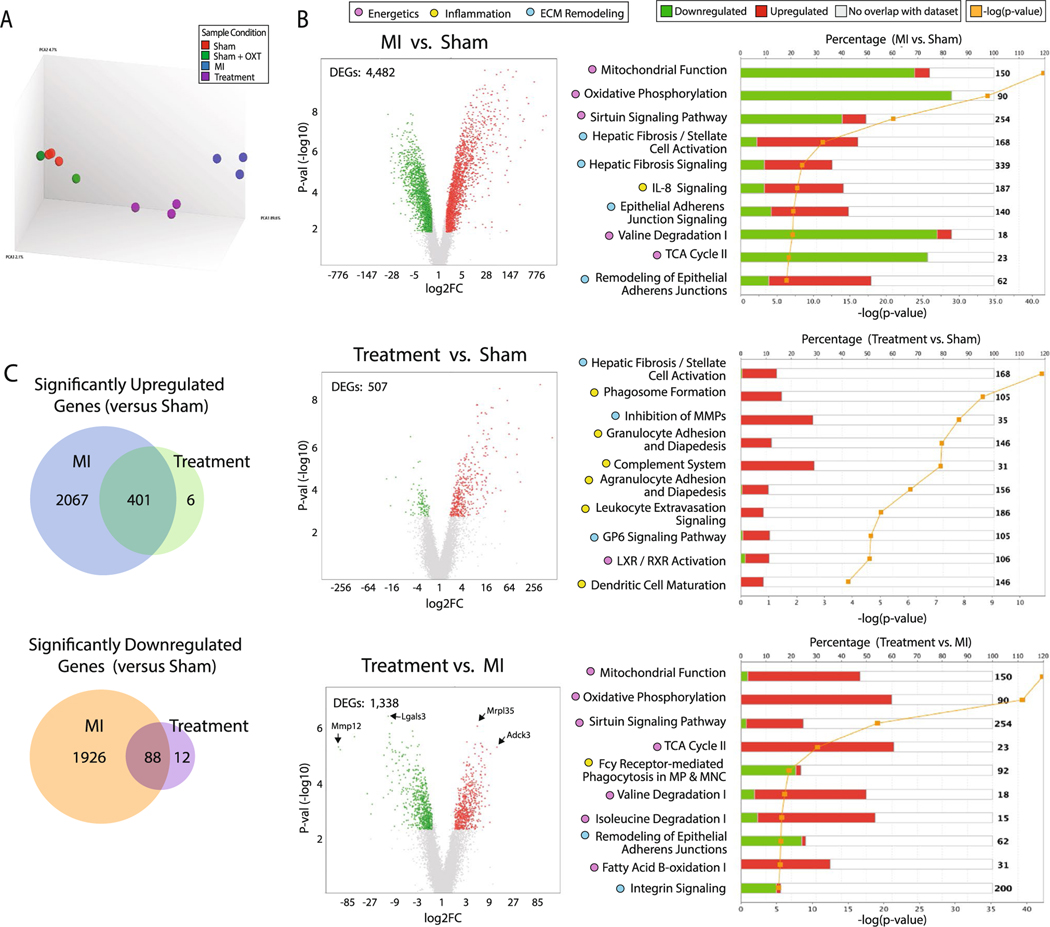
Transcriptome analysis of LV myocardium DEGs. **A** PCA of each group (Sham, *n* = 3; Sham + OXT, *n* = 3; MI, *n* = 3; Treatment, *n* = 3). **B** Volcano plots of DEGs between groups (left; 1.7 < FC < − 1.7, FDR < 0.08), and top ten corresponding differentially regulated canonical pathways represented by the DEGs (right). The stacked bar chart depicts the percentage (upper x-axis) of pathway genes up-, down-, or not differentially expressed (bar color), with the total number of pathway genes shown on the right of each bar, and the -log significance of the differential pathway regulation (orange line; lower x-axis). Only transcripts with FDR < 0.08 were entered into the analysis; all pathways depicted exhibit *p* < 0.05. **C** Venn diagrams of differentially upregulated or downregulated genes compared to Sham. Abbreviations: FC, fold change; FDR, false discovery rate; MI, myocardial infarction; MMPs, matrix metalloproteases; MP, macrophage; MNC, monocyte

To identify potential mechanisms responsible for functional improvements following PVN-OXT neuron activation post-MI, we assessed differential gene expression in the left ventricular (LV) free wall, distal to the left anterior descending (LAD) coronary artery ligation. Raw expression, fold change, and *p* values for all reported genes are provided in Supplemental Data 1. The 23,188 probes present on the Clariom S rat microarray were assessed. A principal component analysis (PCA) showed that Sham and Sham + OXT groups clustered together as one group, as expected. The Sham groups, MI group, and Treatment group all clustered distinctly from one another, indicating that the expression pattern between these three groups was different (Fig. 3A). Transcriptome Analysis Console (TAC; ThermoFisher Scientific) software was used for further analysis, where differentially expressed genes (DEGs) between groups were defined as a fold change (> 1.7 or < − 1.7) that satisfied a false discovery rate of *p* < 0.08. We performed a pairwise analysis of groups for DEGs and visualized upregulated vs. downregulated genes using volcano plots (Fig. 3B). Zero DEGs were identified for the Sham vs. Sham + OXT groups (Fig S1A) so the Sham group was used as the control in subsequent transcriptome analyses. Compared to Sham, the MI group had significantly more DEGs than the Treatment group (19% vs. 2%; 4482 vs. 507 DEGs). Of the 2474 MI DEGs that were upregulated vs. Sham, 401 genes were commonly upregulated between MI and Treatment groups, and of the 2026 DEGs that were downregulated vs. Sham, only 88 were common between MI and Treatment groups (Fig. 3C). The increased number of transcripts that were dysregulated in MI than in Treatment animals suggest that the final injury following LAD ligation was greater in MI than Treatment animals.

Ingenuity Pathway Analysis (IPA; Qiagen) identified pathways that were differentially upregulated or downregulated between groups. The top ten canonical pathways identified are shown in Fig. 3B. When comparing DEGs between Treatment and MI groups, six of the ten top pathways were integral components of cellular or mitochondrial metabolism and were all upregulated in Treatment vs. MI animals. The remaining canonical pathways were related to tissue remodeling and fibrotic processes, which were downregulated in Treatment vs. MI. Integrin and inflammatory signaling were also downregulated in Treatment vs. MI. In the volcano plot of Treatment vs. MI, two of the most significantly downregulated genes were components of matrix remodeling and inflammatory responses, MMP12 and Lgals3, respectively. Mrpl35, which encodes the large mitochondrial ribosomal subunit, and Adck3 (Cox8a), which is integral in mitochondrial respiration, were the top upregulated genes in Treatment vs. MI animals. These findings suggest that chronic PVN-OXT neuron activation after MI may protect mitochondrial function and reduce inflammation, remodeling, and fibrosis.

### Mitochondrial respiration

**Fig. 4:**
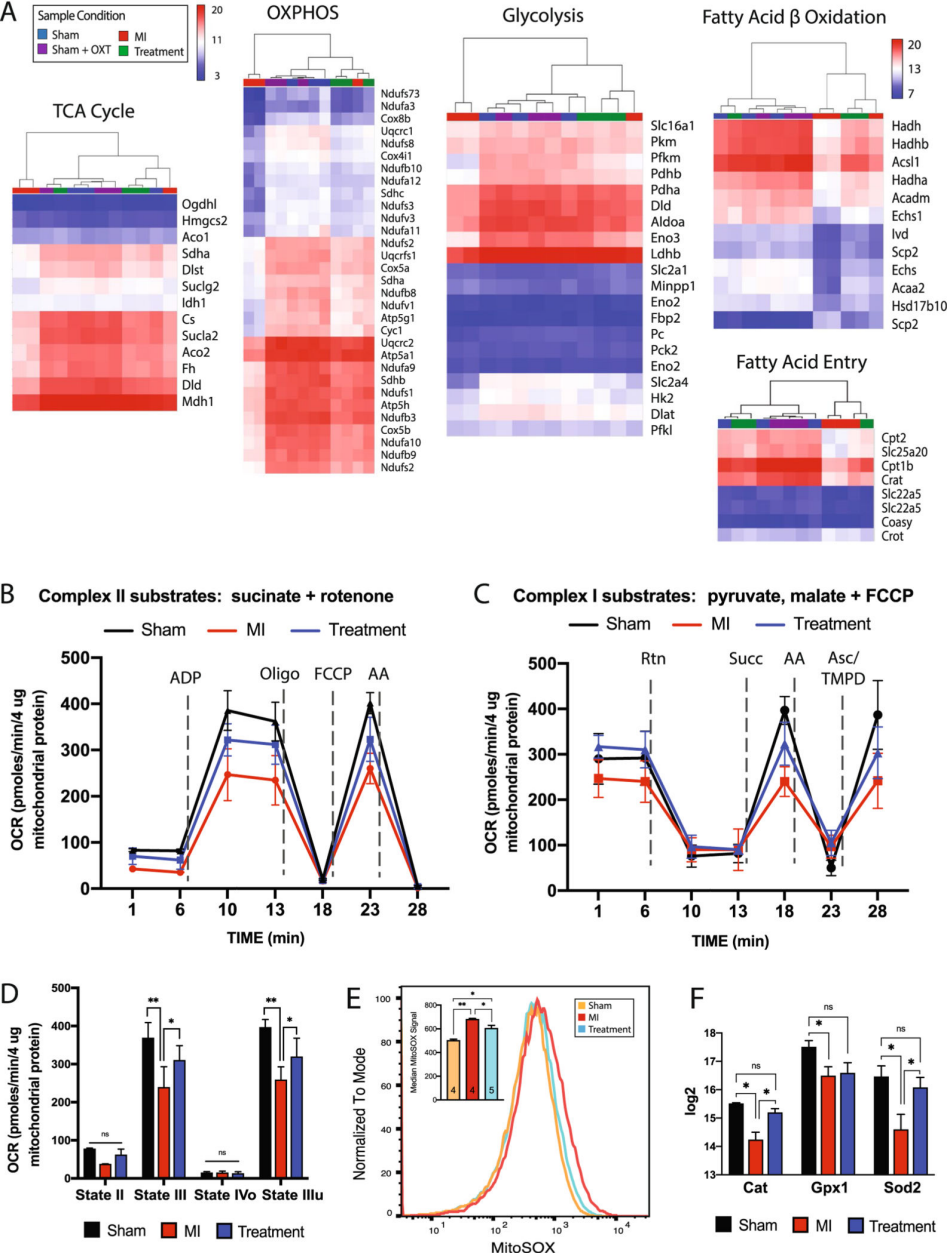
Mitochondrial respiration was preserved in hearts of Treatment animals. **A** Transcriptome expression heat maps and hierarchical clustering of key genes involved in mitochondrial respiration: TCA cycle, OXPHOS, glycolysis, fatty acid beta-oxidation, and fatty acid entry. **B** Seahorse analysis of substrate-stimulated respiration of isolated mitochondria with succinate or **(C)** uncoupled respiration with pyruvate and malate. **D** Quantitation of oxygen consumption rate (OCR) for succinate-stimulated respiration. For all respiration assays: Sham, *n* = 3; MI and Treatment, *n* = 4 each; one-way ANOVA; mean ± SD; *p < 0.05. **E** Flow cytometric analysis of isolated mitochondrial superoxide (mitoSOX; Sham and MI, *n* = 4; Treatment, *n* = 5; one-way ANOVA; mean ± SD; *p < 0.05), and **F** microarray expression of mitochondrial antioxidant enzymes (*n* = 3 per group; one-way ANOVA; mean ± SD; *p < 0.05)

Transcriptome profiling indicated that cellular energetic pathways were significantly altered in Treatment and MI animals, so the expression of enzymes and transporters involved in glycolysis, the tricarboxylic acid cycle (TCA) cycle, oxidative phosphorylation (OXPHOS), and beta-oxidation was analyzed. Hierarchical clustering of essential genes associated with each of these processes suggested a significant reduction in MI vs. Treatment, Sham, and Sham + OXT animals (Fig. 4A). In addition to reduced expression of enzymes involved in cellular respiration, drastically reduced expression of carnitine transporters involved in fatty acid transport was also observed in MI animals.

The reduced expression of mitochondrial genes could be attributed to either decreased global mitochondrial content or a decline in mitochondrial respiratory capacity. This ambiguity was addressed using Seahorse XFe assays of isolated mitochondria. When complex II function was tested, ADP-stimulated respiration (State III) and maximal respiration (FCCP stimulated; State IIIu) were significantly impaired in MI vs. Sham and Treatment animals (Fig. 4B, D). Sequential electron flow through the complexes was also examined (Fig. 4C). Complex-I-stimulated basal respiration was not significantly different between groups, although a trend (*p* = 0.07) of increased oxygen consumption was apparent in Treatment vs. MI animals. Semi-quantitative real-time PCR of Ndufs2, the 49 kDa subunit of complex I, confirmed that expression of this subunit was higher in Treatment animals (Fig S2). Complex-II-driven (succinate) respiration was significantly different between all groups, but complex-IV-stimulated (ascorbate/TMPD) respiration only differed between Sham and MI animals (Fig. 4C). These electron flow assay results support the coupling assay results (Fig. 4B) and suggest that complex II is the most compromised component of the ETC 7 days post-MI and is also significantly protected in Treatment animals.

### Mitochondrial superoxide

Mitochondrial production of reactive oxygen species (ROS) is elevated following MI, with detrimental effects on mitochondrial function [106, 108]. The rate of superoxide production of mitochondria isolated from the LV was measured by flow cytometry to determine if the rate was lower in Treatment animals. Mitochondria that were positive for both mitoTRACKER (live mitochondria) and mitoSOX (superoxide detection) were analyzed. Mitochondria from Treatment animals produced significantly less superoxide radicals compared to those of MI animals (mitoSOX fluorescence of 605 ± 20 and 679 ± 16; *p* = 0.0005) (Fig. 4E). This result was supported by decreased gene expression of the mitochondrial antioxidant enzymes superoxide dismutase (Sod2), catalase (Cat), and glutathione peroxidase in MI animals and their preserved expression in Treatment animals (Fig. 4F). Semi-quantitative real-time PCR of Sod2 confirmed this outcome (Fig. S2).

### Mitochondrial morphology

**Fig. 5:**
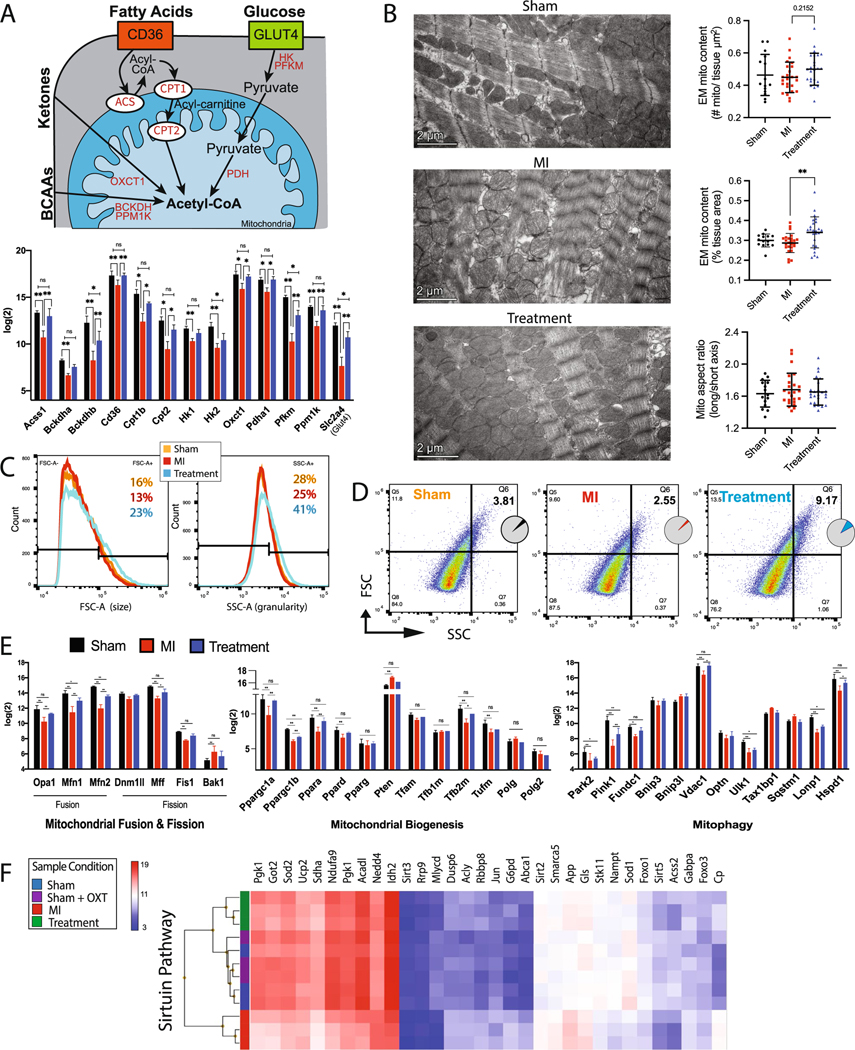
LV mitochondria respiration and morphology were preserved in Treatment animals. **A** Schematic of myocyte substrate utilization and substrate-specific key processes that drive ATP production (top), with expression of genes involved in each component for each group (bottom, *n* = 3 per group; one-way ANOVA; mean ± SD; *p < 0.05). **B** Representative electron micrographs of mitochondrial ultrastructure with derived measurements of mitochondrial content and aspect ratio (*n* = 4 animals per group; one-way ANOVA; **p < 0.01). **C** Representative flow cytometric mitochondrial forward scatter (FSC, left panel) and side scatter (SSC, right panel) depicting size and granularity, respectively. Counts are 50,000 ± 200 mitochondria per group. (Sham and MI, *n* = 4; Treatment, *n* = 5). **D** Representative forward vs. side mitochondrial scatter indicating increased size and complexity (Q6 – top right quadrant) in Treatment animals. **E** Expression of genes involved in mitochondrial fusion and fission (left), biogenesis (middle), and mitophagy (right) (*n* = 3 per group; one-way ANOVA; mean ± SD; *p < 0.05, **p < 0.01). **F** Microarray heat map expression and hierarchical clustering of the sirtuin pathway involved in regulation of mitochondrial dynamics (*n* = 3 per group)

Mitochondrial OXPHOS is governed by the availability of substrate and the pathways that transport and prepare substrates for the TCA cycle (Fig. 5A). The expression of 13 genes (i.e., Glut4, CD36, hexokinase, among others) involved in rate-limiting steps for pathways involving glucose, fatty acids, branched-chain aminos acids (BCAAs), and ketones was compared between groups. The expression of each gene was reduced in MI animals and preserved in Treatment animals (Fig. 5A), with semi-quantitative real-time PCR confirmation of reduced Slc2a4 (Glut4) expression in MI animals (Fig. S2).

Changes in mitochondrial function and ultrastructure are bidirectionally linked. Mitochondrial fusion is largely dependent upon a potential across the inner membrane, suggesting that OXPHOS capacity may affect local mitochondrial size [64]. Additionally, glucose availability, mitochondrial membrane potential, and ROS can differentially impact mitochondrial inner membrane vs. outer membrane fusion [128]. To assess the ultrastructural consequences of reduced substrate pathway gene expression and diminished mitochondrial function (Fig. 4D), mitochondria from the MI border zone and core of the infarct were analyzed by TEM and flow cytometry. TEM examination of border zone tissue revealed no significant differences in the number of mitochondria (per um^2^) between groups (Fig. 5B). Mitochondria were larger in Treatment compared to MI or Sham groups; however, the aspect ratio was no different than Sham, indicating that the mitochondria were not swollen (Fig. 5B). Flow cytometry of MitoTRACKER-positive mitochondria revealed that mitochondria were larger and had increased complexity in Treatment vs. Sham and MI animals (Figs. 5C, D, S4), confirming the TEM results.

Key genes involved in mitochondrial fusion, fission, biogenesis, and mitophagy were differentially expressed between groups (Fig. 5E), with downregulation of genes in MI animals; although Pten, involved in Akt pathway inhibition, and Bak1, associated with apoptosis, were upregulated. The expression of genes associated with fusion (Opa1, Mfn1, and Mfn2) was upregulated in Treatment vs. MI while only the fission gene Mff was upregulated. These results indicate that Treatment mitochondria may respond to injury by promoting fusion while inhibiting fission, thereby attenuating LV dysfunction and infarct size [78, 79]. Genes that regulate mitochondrial DNA synthesis (Polg and Polg2) were not differentially expressed between groups (Fig. 5E).

Another important aspect of mitochondrial regulation is derived from a family of evolutionarily conserved nicotinamide adenine dinucleotide-dependent deacetylases, the sirtuins. Sirtuins (encoded by genes Sirt1–7) regulate mitochondrial protein networks, orchestrate mitochondrial function, and allow cells to adapt to metabolic stress. The sirtuin pathway was one of the top ten canonical pathways dysregulated between MI and Sham animals, with upregulation in Treatment vs. MI, and differential clustering of MI animals apart from Treatment, Sham, and Sham + OXT animals (Fig. 5F). The genes Sirt3, Sirt4, and Sirt5 are localized to the mitochondria and regulate metabolism in response to mitochondrial stress [19, 116, 126]. Sirt3 deacetylates various proteins to regulate amino acid metabolism, fatty acid oxidation, the TCA cycle, the ETC, mitochondrial DNA replication, transcription, and translation. Sirtuins also regulate autophagy, a process by which damaged proteins and organelles are degraded and recycled to provide metabolic intermediates necessary for protein synthesis and metabolism. Maintained expression of key sirtuin genes further establishes that mitochondrial health may have been preserved in Treatment animals compared to MI.

### Immune response

**Fig. 6:**
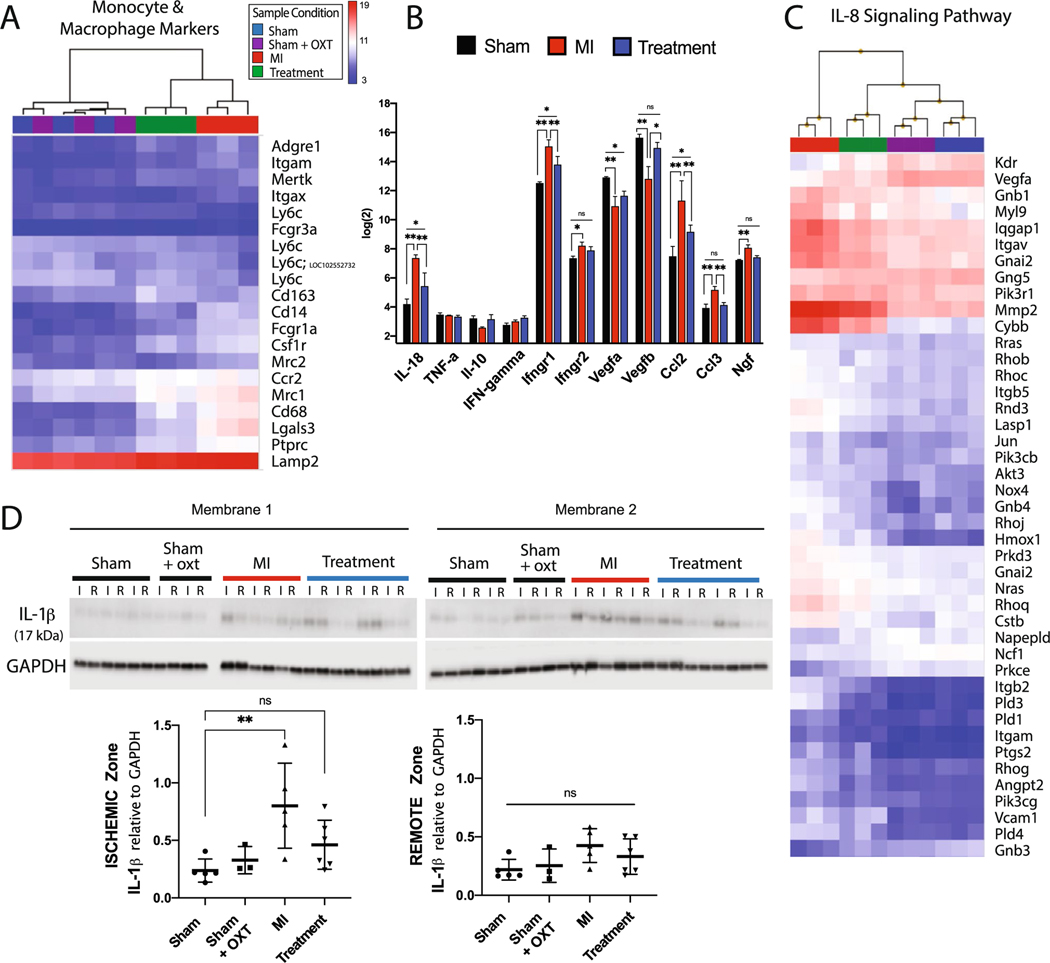
Cardiac inflammation was reduced 7 days after MI in Treatment animals. Microarray expression and hierarchical clustering of **A** cardiac monocyte and macrophage markers, **B** cytokines (*n* = 3 per group; one-way ANOVA; mean ± SD; *p < 0.05, **p < 0.01) and **C** the IL-8 signaling pathway. **D** Western blot of IL-1β expression in ischemic (I) and remote (R) areas of the infarct (Sham, *n* = 5; Sham + OXT, *n* = 3; MI, *n* = 5; Treatment, *n* = 6; one-way ANOVA; mean ± SD, **p < 0.01)

Genes associated with macrophages and monocytes were dramatically increased in MI vs. Sham animals, with a decrease in Treatment vs. MI animals (Fig. 6A). Accordingly, increased gene expression associated with proinflammatory cytokines and chemokines (IL-18 [121], Ifng, Mcp1/Ccl2, Mip-1a/Ccl3) was evident in MI vs. Treatment animals, with increased angiogenic (Vegfa, Vegfb) gene expression in Treatment animals (Fig. 6B). This differential gene expression suggests that in Treatment animals, 7 days post-MI, there was either a timelier repression/resolution of the inflammatory response, or a decreased injury response. This was further supported by a reduction (compared to MI) in Treatment animal gene expression for IL-8 signaling (Fig. 6C), a pathway clinically associated with larger infarct size, lower ejection fraction, and larger increase in LV end-diastolic volume [104]. In contrast, IL-8 signaling was one of the top ten canonical pathways activated in MI animals (Fig. 3B).

The macrophage-secreted cytokine IL-1β is a pivotal cytokine following MI [25]. We found significantly increased levels of the active form of IL-1β in MI vs. Sham animals, yet found no significant elevation in Treatment animals (Fig. 6D). IL-1β and endothelin-1 (Edn1; 3.5-fold increase in MI vs. Sham, 1.4-fold increase in Treatment vs. Sham) have been shown to contribute to the production of nerve growth factor (NGF) [33, 43, 70], and increased NGF is a mechanism responsible for nerve sprouting and sympathetic hyperinnervation following MI [127]. NGF gene expression (Ngf) was significantly elevated in MI but not in Treatment animals (Fig. 6B), aligning with the reduced levels of IL-1β and Edn1 measured for Treatment animals. These immune response observations suggest that MI animals, compared to Sham and Treatment, experienced more severe injury signals after MI and responded with a more robust recruitment of immune cells to the injury, which likely increased pathologic remodeling, autonomic imbalance, and incidence of arrhythmia.

### Fibrosis and matrix remodeling

**Fig. 7:**
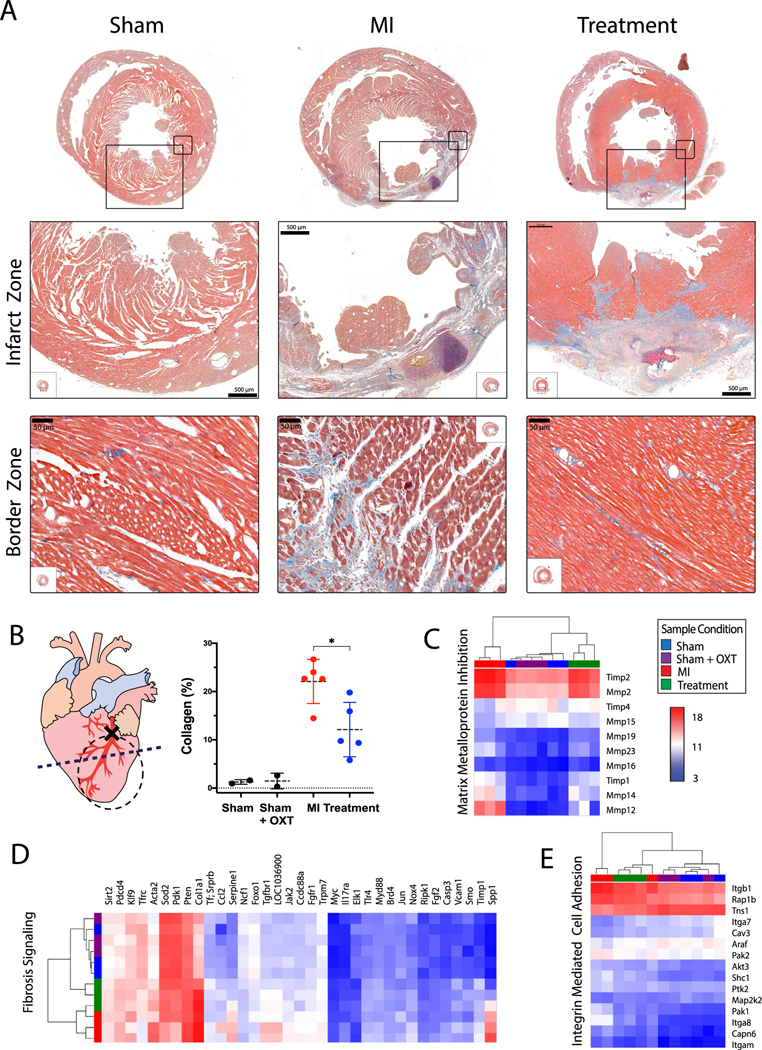
Myocardial remodeling and infarct size were reduced in Treatment animals 7 days after MI. **A** Representative Masson’s trichrome images of myocardial sections from Sham, MI, and Treatment animals are shown in each column. Large and small bounding boxes on images of the full section (top row) indicate the bounding area of the high-resolution images for the infarct zone (middle row) and border zone (bottom row). Blue denotes the presence of collagen. **B** Schematic of the heart (left) illustrates the cross-section of histological assessment (dashed line). The dashed circle represents the location of the ischemic zone (area at risk) from which tissue was harvested for microarray analysis and Western blotting. Percent collagen content (right) within a region of the infarct zone was measured using the trichrome images (Sham, *n* = 3; Sham + OXT, *n* = 2; MI, *n* = 5; Treatment, *n* = 5; one-way ANOVA; mean ± SD; *p < 0.05). Hierarchical clustering and heat map expression of genes involved in **C** inhibition of matrix metalloproteins, **D** fibrosis signaling, and **E** integrin-mediated cell adhesion are provided (*n* = 3 per group)

Ischemic cell death after MI initiates a multiphase reparative response where fibroblasts and myofibroblasts replace damaged tissue with a fibrotic scar. Although the initial reparative fibrosis is crucial for preventing rupture of the ventricular wall, an exaggerated fibrotic response and reactive fibrosis outside the infarct zone may lead to progressive impairment of cardiac function. Trichrome-stained sections containing infarct and border zone tissue illustrated smaller infarcts for Treatment animals, with reduced collagen content in the infarct and border zones (Fig. 7A). Infarct area, quantified as collagen area per area of tissue, was significantly less in Treatment vs. MI animals (12.8 ± 5.6 and ± 4.6, respectively; Fig. 7B). Transcriptome profiling identified that gene transcripts associated with LV remodeling and fibrosis were markedly increased 7 days after MI. Four of the top ten canonical pathways represented by pairwise analysis of MI vs. Sham DEGs were related to LV remodeling (Fig. 3B), including hepatic fibrosis/stellate cell activation, hepatic fibrosis signaling (Fig. 7D), epithelial adherens junction signaling, and remodeling of epithelial adherens junctions (Fig. 7E). Semi-quantitative real-time PCR confirmed increased presence of Mmp12 in MI vs. Sham and Treatment animals (Fig S2). Treatment animals also had significantly fewer DEGs associated with LV remodeling and fibrosis, and genes that inhibit matrix metalloproteins were upregulated (Figs. 3B and 7C).

### ECG morphology and arrhythmias

**Fig. 8:**
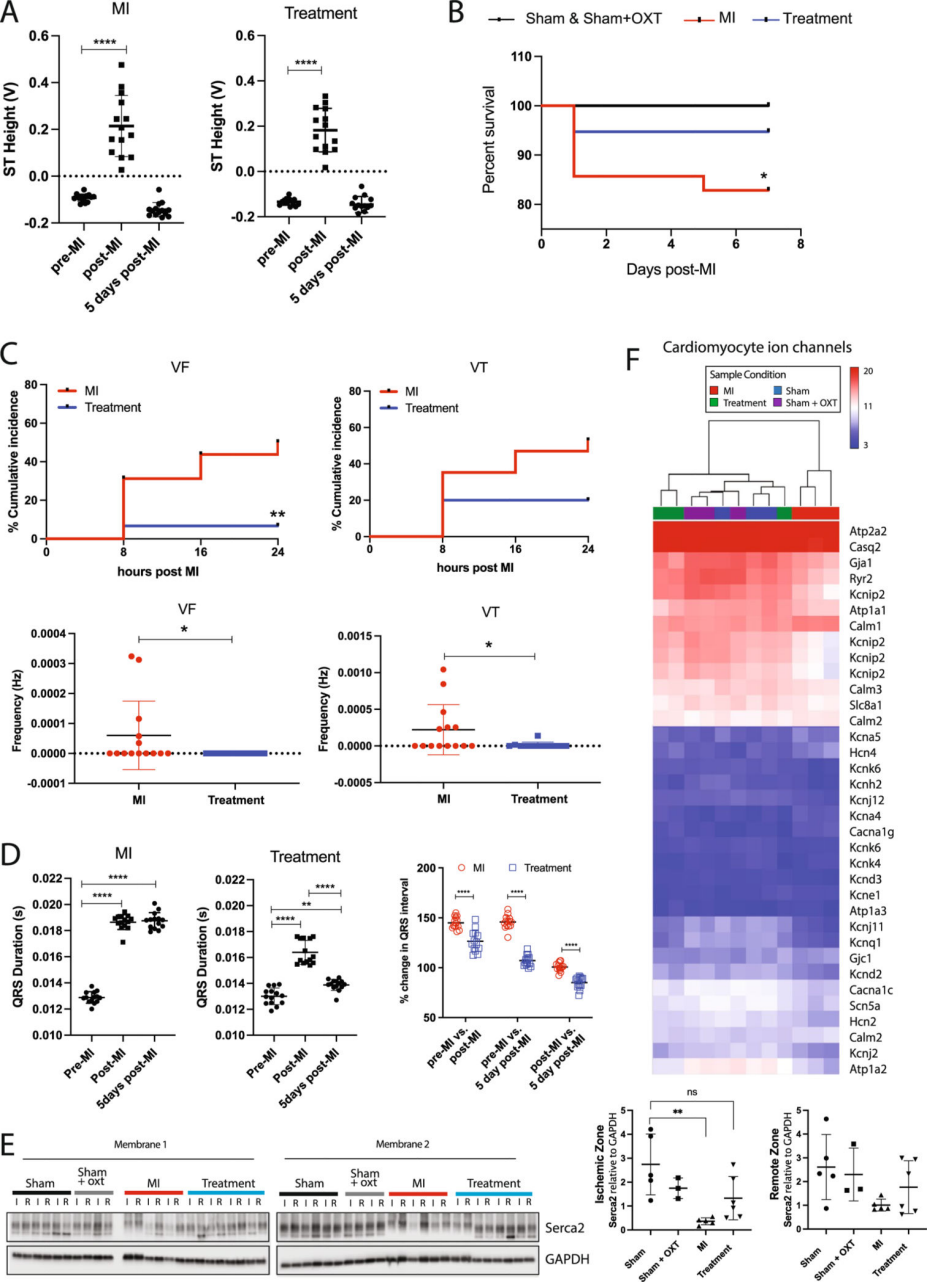
Arrhythmia incidence and mortality were reduced in Treatment animals. **A** Average 24 h ST segment elevation immediately following MI and 5 days post-MI is similar between MI and Treatment animals, indicating that both groups experienced a similar degree of ischemic damage. Pre-MI: MI, *n* = 12; Treatment, *n* = 13. Post-MI: MI, *n* = 14; Treatment, *n* = 13. Five days post-MI: MI, *n* = 14; Treatment, *n* = 15. One-way ANOVA; ****p < 0.0001. **B** Post-MI survival is significantly improved in Treatment animals compared to MI animals (Sham, *n* = 25; MI, *n* = 35; Treatment, *n* = 38; Kaplan–Meier; *p = 0.046). **C** In the 24 h immediately following MI, Treatment animals had lower incidence of arrhythmias (MI, *n* = 14; Treatment, *n* = 12; unpaired *t* test; **p < 0.01, *p < 0.05) *VF* ventricular fibrillation, *VT* ventricular tachycardia. **D** QRS duration significantly increased after MI in both groups but was significantly lower 5 days post-MI in Treatment animals. Multiple comparisons of percent increases in QRS duration from pre-MI values reveal that QRS widening was also significantly less for Treatment animals compared to MI animals (same animal numbers as panel A, one-way ANOVA, **p < 0.01, ****p < 0.0001). **E** Western blots for Serca2a (Atp2a2) expression of LV tissue (I: ischemic) and RV tissue (R: remote) 7 days after MI are shown (left). Serca2a was reduced in the I zone for MI animals but not Treatment animals (right, Sham, *n* = 5; Sham + OXT, *n* = 3; MI, *n* = 5; Treatment, *n* = 6; one-way ANOVA; mean ± SD, **p < 0.01). **F** Expression and hierarchical clustering of genes that are key contributors to the cardiac action potential. MI animals cluster alone yet Treatment animals cluster with Sham and Sham + OXT animals, indicating preserved expression of key genes in Treatment animals (*n* = 3 per group)

Immediately following MI surgery, MI and Treatment animals displayed similar magnitudes of ST elevation, confirming a similar degree of ischemic damage after LAD ligation in both groups (Fig. 8A). The ECG was continuously recorded during the first 24 h after MI surgery in consideration of the high incidence of arrhythmia and sudden cardiac death (SCD) during this period [50]. Within the first 24 h, survival rate dropped to 85% and 95% for MI and Treatment animals, respectively (Fig. 8B), with Treatment animals having significantly lower cumulative incidence and frequency of VF and VT compared to MI (Fig. 8C). Ischemic arrhythmia mechanisms during the acute phase of infarction are multifaceted and include substrate alterations [50, 57, 111, 130] (depolarized resting membrane potential, increased dispersion of refractoriness, and slowed conduction) that disrupt excitation wavefronts and initiate reentry [73, 87], triggered electrical activity [55, 93, 122], and increased catecholaminergic activity [16, 61]. Compared to pre-MI, QRS duration for MI and Treatment animals was significantly longer in the first 24 h post-MI, suggesting reduced repolarization currents or slowed ventricular conduction (Fig. 8D). At 5 days post-MI, this QRS prolongation persisted for MI but was significantly less for Treatment animals Fig. (8D).

At 7 days after MI, we observed differential gene expression for many proteins that regulate cardiomyocyte excitation and contraction (Fig. 8E, F). While at the transcript level, Atp2a2 showed minor changes, Serca2a protein level was significantly lower in the ischemic (I) zone for MI vs. Sham animals yet there was no difference in Serca2a protein level for Treatment vs. Sham (Fig 8E), indicating a potentially higher risk for Ca^2+^-mediated triggered arrhythmias in MI animals. Expression for proteins responsible for excitation–contraction coupling (Atp2a2/Serca2a, Cav1.2/Cacna1c, Ryr2) was dysregulated in MI animals (Fig. 8F), consistent with pathological remodeling associated with MI and progression to heart failure.

Altered resting membrane potential will promote spontaneous electrical activity and arrhythmias. Several classes of genes for proteins that maintain resting membrane potential were downregulated in MI vs. Sham and Treatment animals. For example, gene expression for inward rectifier K + channel subunits (Kir2.1/Kcnj2, Kir6.2/Kcnj11, Kir2.2/Kcnj12) and the Na + /K + ATPase (Atp1a2) was reduced. The ratio of transcripts for the funny current main subunits Hcn2/Hcn4 was also reduced, consistent with myocyte reversion to a more immature phenotype [105]. Altogether, these changes increase the propensity for ectopic activity and arrhythmia.

Transcriptome profiling also indicated dysregulation of myocyte excitability and repolarization. Isoform gene expression of Kcnip2, a purported master transcriptional regulator of cardiac excitability [84], was reduced in MI vs. Sham and Treatment animals, as was expression for the main depolarizing ion channel, the voltage-gated Na + channel (Nav1.5/Scn5a) (Fig. 8F). Prolonged QRS duration in MI vs. Sham and Treatment could be the result of reductions in the rapid delayed rectifier K + channel Kv11.1/Kcnh2 and reduced cellular coupling via gap junctions, as indicated by lower expression of Gja1 (Connexin 43) (Fig. 8F), a result confirmed by semi-quantitative real-time PCR (Fig S2). Such altered expression of sarcolemmal proteins likely contributed to a pro-arrhythmic increase in dispersion of repolarization and slowed conduction in MI animals. In total, these results support increased prevalence of arrhythmia mechanisms in MI animals that involve altered membrane excitability, increased triggered activity, increased dispersion of repolarization, and slow conduction.

## Discussion

Altered autonomic balance, with increased sympathetic drive and decreased parasympathetic tone, is a hallmark of cardiovascular disease including MI, heart failure, sleep apnea, and diabetes [22, 31, 110, 115, 118]. Although implantable device-based activation of parasympathetic drive provides potent cardioprotection during disease, there is no non-invasive rapid approach for parasympathetic activation during an acute MI. Our previous work has shown that CVNs receive powerful excitation from a population of hypothalamic PVN-OXT neurons that co-release OXT and GLUT to excite CVNs [28, 49, 92]. Activation of those PVN-OXT neurons reduces blood pressure and heart rate in conscious unrestrained animals, and those effects are para-sympathetically mediated [34, 97]. In this report, we present new evidence that activation of PVN-OXT neurons soon after an MI is cardioprotective during the 7 days following an MI. We also provide new insight into the molecular basis of the multifaceted aspects underlying that cardioprotection.

### Neurotransmission

The present study underscores the profound bidirectional interaction between the central nervous system and the heart after an MI. We found that an MI results in diminished excitatory neurotransmission from PVN-OXT neurons to brainstem parasympathetic neurons, leading to reduced cardioprotection. We also demonstrated that daily activation of PVN-OXT neurons maintains excitatory neurotransmission to brainstem parasympathetic neurons and sustains the expression of muscarinic receptors (Chrm2) within the myocardium, thereby supporting muscarinic-mediated cardioprotective outcomes of the autonomic parasympathetic network.

LV gene expression analysis revealed that activation of PVN-OXT neurons after an MI maintained signaling pathways and transcriptional responses that are protective against myocardial injury. As indicated in Fig. 2C, many of the cardioprotective effects of parasympathetic drive are due to post-ganglionic release of ACh and subsequent activation of inhibitory pathways within myocytes, which was potentially mediated in Treatment animals by increased expression of Chrm2. This result is consistent with others who have reported increased Chrm2 expression in HF rats treated with carvedilol (an α- and β-blocker), indicating that the upregulation of muscarinic receptors is consistent with cardioprotection [124]. Long-term treatment with carvedilol also restored autonomic tone in patients with moderate HF [77]. Furthermore, multiple pre-clinical studies have shown cardioprotection following ischemia/reperfusion injury by activating cholinergic muscarinic receptors (mAChRs), as well as nicotinic receptors (nAChRs), either pharmacologically or by direct-current electrical stimulation [45].

Expression of the muscarinic M2 receptor via Chrm2 transcriptional activity is tightly regulated by the gene silencing transcription factor Rest/Nrsf (RE-1 silencing transcription factor), which may act via epigenetic remodeling to repress neural genes in non-neural cells [42, 132]. Rest expression was significantly upregulated (2.1-fold) in MI vs. Sham animals (FDR *p* = 0.01) but was non-significantly downregulated (1.1-fold) in Treatment vs. Sham animals. This suggests that excitatory signals mediated by PVN-OXT neuron activity may alter Chrm2 abundance through epigenetic actions of Rest/Nrsf. Additionally, Rbm24, an RNA binding protein that drives various post-transcriptional processes and is known to interact with Chrm2 transcript [69], was reduced after MI yet preserved in Treatment animals. Consistent with altered Chrm2 expression in the heart [38], we found that HRR was longer in MI animals while Treatment animals had shorter HRR times that were no different than Sham (Fig. 2E). HRR after peak effort is a common assessment of autonomic balance following adverse cardiovascular events, with a longer HRR time associated with increased mortality, sudden cardiac death, and arrhythmic events [53, 85, 109]. As such, it was not unexpected to find that the untreated MI animals had longer HRR times and higher mortality than animals treated with PVN-OXT neuron activation (Fig. 8B). Consistent with our results, work from others has shown pyridostigmine bromide, a reversible anticholinesterase agent that exerts cholinergic stimulation, improves HRR after exercise [4], increases heart rate variability, and decreases the density of ventricular arrhythmia in patients with heart failure [7].

### Arrhythmia incidence

In the first 24 h following LAD ligation, both MI and Treatment animals exhibited similar ST segment elevations, indicating a similar level of myocardial injury. In MI animals, there were frequent bursts of arrhythmia, including VF and VT, that often occurred in the first hour after LAD ligation. In Treatment animals, arrhythmias in the 24 h after LAD ligation were significantly less frequent, shorter in duration, and often absent (Fig. 8C). This is reflected in the increased survival of Treatment animals compared to MI (Fig. 8B). The higher mortality of MI animals is consistent with the lower EPSC amplitudes of the parasympathetic DMNX neurons observed for that group (Fig. 2A, B) and reports of reduced vagal drive to the heart being a strong independent risk factor for life-threatening arrhythmias and sudden cardiac death [9]. The reduced incidence of arrhythmias for Treatment animals is consistent with the cardioprotective effects of cholinergic muscarinic activation, as reported during ischemia/reperfusion injury, where hearts pretreated with choline had significantly decreased ischemia-induced arrhythmia, fewer ventricular premature beats, and a smaller infarct size [23, 133, 134]. Furthermore, expression of key genes responsible for cardiac excitation and contraction, such as connexin 43 (Gja1), Nav1.5/Scn5a, Cav1.2/Cacna1c, repolarizing K + channels, and the ryanodine receptor (Ryr2), were likewise preserved in Treatment animals compared to MI (Fig. 8F). Significant preservation of ischemic zone Serca2 protein expression in Treatment animals at a level similar to Sham (Fig. 8E) indicates maintenance of the capacity of the sarcoplasmic reticulum to sequester Ca^2+^.

Our observations of reduced arrhythmia incidence and the associated preserved expression levels of key genes during PVN-OXT neuron activation in animals with an acute MI are supported by previous studies of electrical vagal nerve stimulation (VNS) during MI. In one study, VNS demonstrated reduced incidence of VF during coronary artery occlusion in canines [27]. A more recent study found that chronic VNS, applied after permanent MI in Yucatan minipigs, stabilized the LV scar-border zone by reducing heterogeneity in activation and repolarization *in vivo*, drastically reducing lethal ventricular arrhythmias [36]. Although shown to be beneficial in controlled experiments, VNS devices are not selective for cardiac cholinergic fibers and implanting the devices before, or at the onset of, unanticipated episodes of cardiac ischemia, and other triggers of sudden cardiac death, is not clinically feasible [17]. A recent clinical study demonstrated that low-level tragus stimulation reduced the incidence of reperfusion-related ventricular arrhythmias during the first 24 h after acute MI [129]. Although highly encouraging, studies of human anatomy found that tragus distribution of the auricular branch of the vagus nerve is present in only 45% of the cases [89], possibly limiting the efficacy of low-level tragus stimulation in patients with different nerve supplies of the tragus [40].

### Mitochondria

Ischemia causes mitochondrial function and structure alterations that impair ATP production and increase ROS production [10, 13, 62, 112]. In healthy myocardium, substrate utilization is tightly regulated to meet changes in energy demand and this metabolic regulation is impaired by ischemia and disease [44]. This involves reduced contribution of fatty acid oxidation to energy production and increased glycolysis, as described for hypertrophied and failing hearts [3, 120]. Increasing evidence suggests that the loss of metabolic substrate flexibility is a major contributor to the development of cardiac dysfunction and heart failure [59].

We found that many genes for rate-limiting proteins integral for substrate utilization were reduced in MI and preserved in Treatment animals (Fig. 5A), suggesting that chronic PVN-OXT neuron activation supported the maintenance of metabolic flexibility after an MI. Furthermore, MI animals had reduced expression of crucial enzymes and intermediates involved in glycolysis, the TCA cycle, OXPHOS, and fatty acid beta-oxidation (Fig. 4A), and this was attributed to a decline in mitochondrial quality (Fig. 5B–D) and respiratory capacity rather than decreased mitochondrial content at 7 days post-MI. These results were confirmed by mitochondrial XF assays where we found that complex I and II of the ETC were compromised in response to MI, with complex II impacted more dramatically, and that the function of complex I and II was significantly protected in Treatment animals (Fig. 4B–D). Similar mitoprotection and fuel preference restoration have been observed in response to VNS following MI [74], isoproterenol-induced ischemia [125], and ischemia/reperfusion injury, and appears to be mediated through efferent fiber activation which is consistent with our treatment paradigm [86].

The most differentially regulated upstream pathway between MI and Treatment groups identified by IPA was peroxisome proliferator-activated receptor gamma coactivator 1-alpha (PGC-1⍺; Ppargc1a), with activation in Treatment and repression in MI animals (Fig. 5E). PGC-1⍺ is a transcriptional coactivator that regulates metabolic genes and is the master regulator of mitochondrial biogenesis [26, 27]. The mitochondrial deacetylase, sirtuin 3 (Sirt3), functions as a downstream target gene of PGC-1⍺ and mediates fatty acid metabolism [41], mitochondrial quality control and dynamics [102], and is postulated to regulate flux through the TCA cycle [117]. Evidence suggests that Sirt3 also regulates the mitochondrial unfolded protein response and acts to sort moderately stressed from irreversibly damaged organelles by activating antioxidant machinery or mitophagy [60, 88]. We found that Sirt3 gene expression was preserved in Treatment animals with a concomitant reduction in mitochondrial unfolded protein response and endoplasmic reticulum stress response (IPA results not shown). This is consistent with the preserved structure and function of our Treatment mitochondria (Figs. 4D, 5B) and prior reports of preserved endothelial cell mitochondria and endoplasmic reticulum after administering ACh following hypoxia/reoxygenation injury[8, 123].

A mechanism of Sirt3-mediated cardioprotection is the preservation of Opa1 gene expression. Opa1 encodes a protein that is critical for inner mitochondrial membrane fusion and the maintenance of proper cristae structure [102]. In addition to Opa1, Treatment vs. MI animals had preserved expression of key mitochondrial genes involved in mitochondrial fusion and fission, biogenesis, and mitophagy (Fig. 5E). Preservation of the aforementioned mitochondrial dynamics has been observed in multiple reports using direct ACh application, m2AChR agonist, and VNS, and attributed to preserved cell survival and function [107, 113]. Consistent with these findings, mitochondrial ultrastructure as well as flow cytometric forward and side scatter analysis suggested that Treatment mitochondria were larger, more granular, and maintained their elongated shape, further supporting improved mitochondrial dynamics and OXPHOS in Treatment animals (Fig. 5C, D). Although the specific mechanisms for the preservation of mitochondrial structure and function conferred by PVN-OXT neuron activation after an MI remain to be rigorously tested, activation of Akt and AMPK (Fig. 2C) are likely candidates because these kinases have been implicated in the prevention of mitochondrial dysfunction by electronic VNS after ischemia/reperfusion injury [6, 86, 106, 125]. Altogether, our results demonstrate that the outcomes of PVN-OXT neuron activation immediately after an MI may include the preservation of mitochondrial OXPHOS, maintained mitochondrial fusion and biogenesis, reduced mitochondrial ROS, and conserved sirtuin pathway signaling components. These functional outcomes are supported by preserved expression of numerous genes involved in mitochondrial structure and function, and provide new molecular insight into potential cardioprotective pathways.

### Inflammation

Although a temporary inflammatory response after MI is required to clear the myocardium of cellular debris and toxic metabolites, excessive chronic inflammation leads to adverse LV remodeling and heart failure. During MI, neutrophils, followed by Ly6C^high^ (Ly6c) mononuclear cells, are quickly recruited to the infarct [32]. Circulating monocytes, upon entry into tissues, give rise to dendritic cells and macrophages. Macrophages phenotypically differ from monocytes by increased expression of Cd68 as well as F4/80 (Adgre1). These monocyte-derived macrophages produce both pro-inflammatory and anti-inflammatory mediators (cytokines, chemokines, matrix metalloproteinases, and growth factors), phagocytize dead cells, and promote angiogenesis and scar formation.

Seven days after MI, leukocyte CD45 (Ptprc) marker expression and markers for cardiac macrophages and monocytes were dramatically elevated in MI vs. Sham animals, with a corresponding decrease in Treatment vs. MI animals (Fig. 6A). Increased expression of pro-inflammatory cytokines and chemokines was evident in MI vs. Treatment animals, with increased angiogenic gene expression in Treatment animals (Fig. 6B), suggesting either a timelier resolution of inflammation or reduced injury response in Treatment animals. These results are consistent with differences between MI and Treatment animals in ischemic zone collagen content (Fig. 7B) and 24 h arrhythmia burden (Fig. 8C), aligning with studies that identified a positive correlation between systemic inflammation in the first 5 days after MI with the size of the peri-infarct zone [94] and the incidence of ventricular arrhythmias [52].

Cholinergic anti-inflammatory pathways are potently activated by electrical VNS, as demonstrated in previous studies that prevented the release of pro-inflammatory cytokines such as TNF-⍺, IL-1β, IL-6, and IL-18 during endotoxemia [11], and in patients receiving tragus stimulus following acute MI [129]. In other studies, activation of cholinergic anti-inflammatory pathways through electrical VNS or muscarinic receptor agonists promoted macrophage M1 to M2 polarization in ischemic heart and lung injury [20, 66], with AMPK signaling as a central regulator of the response [101]. Accordingly, in Treatment animals, we found increased M2-type reparative markers (Cd163, IL-10, Ly6c) and a decrease in pro-inflammatory M1 markers, cytokines, and chemokines (Mcp1/Ccl2, Mip-1a/Ccl3, Cd68) (Fig. 6A, B), suggesting that PVN-OXT neuron activation may promote timely inflammatory resolution and promotion of wound healing and tissue repair.

IL-1β protein was elevated in MI vs. Sham animals with no significant elevation in Treatment animals (Fig. 6D). This macrophage-secreted cytokine was shown to induce arrhythmias in metabolically compromised mice [82] and is a pivotal cytokine in neuroinflammation following MI [25]. Endothelin-1, and IL-1β contribute to the production of NGF in the rodent heart [1, 43], the pulmonary bronchi [33], and the non-neuronal cells of the sciatic [70]. Increased NGF is one of the immune-stimulated mechanisms responsible for nerve sprouting, sympathetic hyperinnervation, and pathological rise in sympathetic activity following MI [127]. Regions of denervation and hyperinnervation may lead to heterogeneity of sympathetic nerve distribution and contribute to cardiac arrhythmias [39]. Interestingly, previously identified underlying mechanisms of VNS-mediated electrical stability include suppressing cardiac neuronal sprouting, inhibiting excessive sympathetic nerve sprouting, and proinflammatory response by regulating gene expression [36, 135]. Accordingly, Ngf transcript was significantly elevated in MI but not in Treatment animals.

Additionally, IL-8 signaling was one of the top ten canonical pathways activated in MI animals but was significantly reduced in Treatment animals. IL-8 pathway activation is clinically associated with larger infarct size, lower LV ejection fraction, larger increase in LV end-diastolic volume, and higher frequency of microvascular obstruction [104]. Overall, these inflammatory outcomes suggest that during MI, untreated animals, compared to treated animals, may experience more severe tissue injury signals with a corresponding robust and sustained recruitment of immune cells to the injury, which likely increased pathologic structural remodeling and the incidence of arrhythmia.

### Structural remodeling

Gene transcripts contributing to LV remodeling were markedly increased following MI, with Treatment animals having significantly fewer DEGs associated with fibrosis and an upregulation of genes that inhibit matrix metalloproteins (Figs. 3B and 7C). Infarct area, measured as collagen area per area of tissue, was also significantly greater in MI vs. Treatment animals. Activation of CVNs has been shown to reduce MI size, not only through heart rate reduction, but through a number of mechanisms including attenuated formation of reactive oxygen species and inflammation, and improved mitochondrial function [40]. Previous studies indicate that a lack of cardiomyocyte-secreted ACh can cause maladaptive remodeling and cardiac functional decline [96, 100] and that over-expression of cardiomyocyte vesicular ACh transporter or choline acetyltransferase increases ACh synthesis which then inhibits ventricular remodeling [99]. Cholinesterase inhibitors, such as donepezil, are also known to improve autonomic balance, and can reduce myocardial infarct size and arrhythmia, and improve LV function following ischemia–reperfusion injury [56]. Interestingly, in many studies utilizing VNS, the infarct limiting effect is only observed when VNS is applied during ischemia, but not at the onset of reperfusion [17, 106]. Subsequent studies have shown that activation of cardiac Chrm2 receptors also exerts an infarct limiting effect [67, 90]. A proposed mechanism of these beneficial outcomes is the inhibition of endoplasmic reticulum stress-induced apoptosis through extracellular signal-regulated kinase (ERK1/2) and the phosphoinositide 3-kinase (PI3K)/protein kinase B (Akt) signaling pathway in combination with the inhibition of adenylyl cyclase activity via Gαi of the m2AChR, thereby reducing cAMP production and further attenuation of ER-stress and apoptosis (Fig. 2C) [67].

In addition to OXT network activation and VNS, parasympathetic-mediated cardioprotection, particularly following ischemia reperfusion, is likely complex and may involve both vagus-dependent and independent mechanisms. Protection independent of the vagus may be mediated by GLP-1 receptors that act via M3 muscarinic receptor activation [5]. Consistent with the potential benefit of peripheral muscarinic receptor activation post-ischemia–reperfusion, studies have shown that acetylcholine activation of α7 nicotinic receptors (α7nAChR) on macrophages polarizes the pro-inflammatory into anti-inflammatory subtypes, activating the transcription 3 (STAT3) signaling pathway, inhibiting the secretion of pro-inflammatory cytokines, limiting ischemic injury in the myocardium, and initiating efficient reparative mechanisms [20]. Furthermore, vagus-mediated ischemic preconditioning and cardioprotection may involve release of humoral factors which subsequently act on many downstream vagal targets and function [58], including a vago-splenic axis [68]. Clinical studies suggest remote ischemic conditioning-induced cardioprotection likely involves activation of sensory nerve fibers [81], while acute caffeine intake can possibly provide a cardioprotective effect through increased vagal tone [103]. In a mouse model of spared nerve injury (SNI) neuropathic pain, myocardial infarct size and apoptosis were reduced following MI, and this protection was dependent upon activation of the para-ventricular thalamus and the autonomic nervous system, as shown by loss of SNI-induced cardioprotection by parasympathetic nerve blockers [18].

## Conclusion

These comprehensive results demonstrate that daily activation of PVN-OXT neurons, beginning soon after an MI, could provide potent cardioprotection against the deleterious effects of MI. PVN-OXT neurons and potentially other approaches to activate the oxytocin network may, therefore, be promising therapeutic targets to quickly activate beneficial parasympathetic-mediated cellular pathways within the heart during the very early stages of infarction. These findings are highly translational as our clinical work in patients with obstructive sleep apnea demonstrates that intranasal oxytocin administration is beneficial, in part, by increasing cardiac parasympathetic activity [47, 48].

### Limitations

We studied a rat model of acute MI induced by permanent coronary occlusion, which has relevance to STEMI patients who do not receive timely (~ 25% of patients [72]) or successful (~ 30% of patients [95]) reperfusion. While both reperfused and non-reperfused MI animal models are clinically relevant [12, 71], our model represents a smaller proportion of patients. Even so, effective, rapid, and easy to administer treatments are needed to reduce mortality during and after an acute MI, even if the occlusion is subsequently removed. Therapies that demonstrate efficacy in reducing arrhythmia burden, inflammation, and infarct size would be relevant for MI at any early stage, including in-transit to the clinic to receive PCI, and even after PCI when incomplete reperfusion may result in regions having permanent lack of flow. Molecular pathways activated in response to permanent occlusion, as we have described, could be different than those activated by ischemia reperfusion injury. Identifying the benefits of PVN-OXT neuron activation after ischemia reperfusion injury is the subject of future studies.

Transcriptomic analysis is a powerful tool, yet differential mRNA expression does not always translate to differential protein expression or activity. In addition to epigenetic modifications, the transcriptional response is the initial cellular response to a stimulus, while the protein response could be altered by many post-transcriptional and post-translational mechanisms. We have reported primarily transcriptional alterations with protein confirmation of only key genes that are known to be regulated post-transcriptionally [119]. An analysis of protein levels associated with other differentially expressed genes and their active state would provide deeper insight into the many molecular mechanisms by which PVN-OXT neuron activation imparts its cardioprotective effects, including its impact on non-neuronal cholinergic system dynamics, remodeling of neuronal circuitry, and infarct limiting effects, which are the focus of future work.

Another limitation of the current work is that we activated the PVN-OXT network in the CNS using chemogenetics. Further work will need to explore the efficacy of activating the OXT network in the CNS with intranasal oxytocin (or other approaches), as well as exploring the role of oxytocin receptors outside the CNS, including oxytocin receptors in cardiac tissue and sensory neurons [35].

## Supplementary Material

Supp Mat #1

Supp Mat #2

## Figures and Tables

**Table 1:** Animal numbers for each experimental group for each assessment

Assessment	Sham	Sham + OXT	MI	Treatment

Post-MI survival	13	12	35	38
24 h ECG pre-MI (ST and QRS analyses)	–	–	12	13
24 h ECG post-MI (ST and QRS analyses)	–	–	14	13
24 h ECG 5 days post-MI (ST and QRS analyses)	–	–	14	15
Incidence of VT and VF analysis	–	–	14	12
Excitatory post-synaptic current measurements*	11	–	6	5
HRR assessments	24	–	9	13
Mitochondrial respiration measurements	3	–	4	4
Mitochondrial ROS assay	4	–	4	5
Mitochondrial scatter assay	4	–	4	5
Western blot of IL-1β	5	3	5	6
Western blot of Serca2a	5	3	5	6
Masson’s trichrome histology of collagen	3	2	5	5
EM assessments of mitochondria structure	4	–	4	4
Microarray transcriptomics (all expression profiles)	3	3	3	3

* for the excitatory post-synaptic current measurements, Sham: *n* = 11 animals, 24 brainstem slices, 24 cells, MI: *n* = 6 animals, 17 brainstem slices, 17 cells; Treatment: *n* = 5 animals, 14 brainstem slices, 14 cells

## Data Availability

The authors declare that the data supporting the findings of this study are available within the paper and its Supplementary Information files. Any remaining data that support the results of the study will be available from the corresponding authors upon reasonable request. Source data are provided within this paper.
