# Supplementary material for: Outcomes of hypothalamic oxytocin neuron-driven cardioprotection after acute myocardial infarction
Source: Basic Res Cardiol. Author manuscript; Available in PMC 2023 Oct 24. (PMC10558415; doi:10.1007/s00395-023-01013-1)

Supplemental Figures

Supplemental figure 1

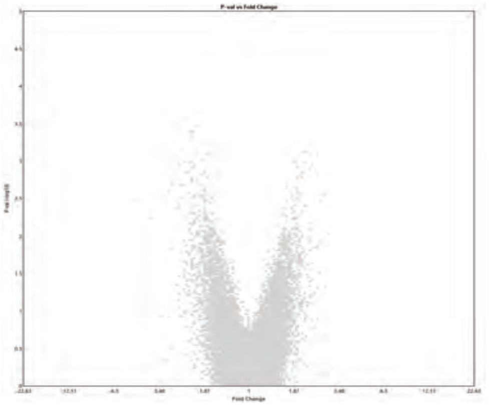

Supp fig 1. Volcano plot of DEGs between Sham and Sham + OXT groups.

Supplemental figure 2

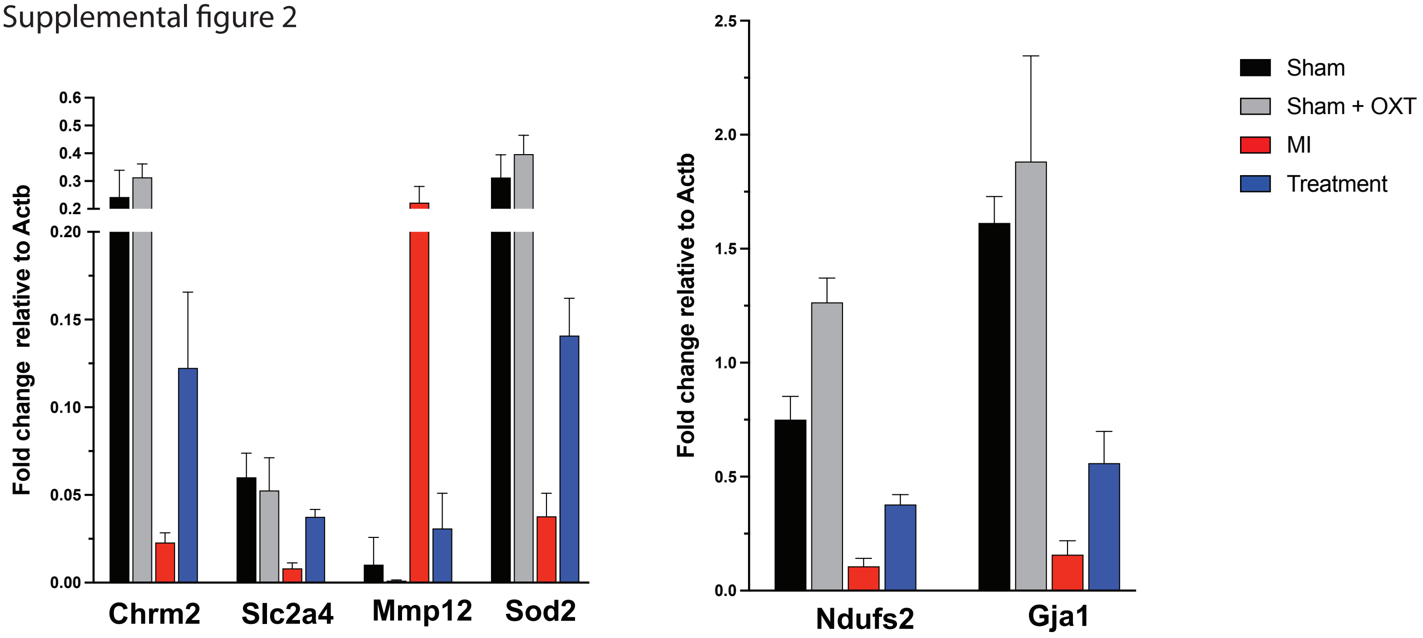

Supp fig 2. Semi-quantitative rt-PCR of select DEGs confirm microarray expression fold changes.

Supplemental figure 3

Gene	Forward (5' --> 3')	Reverse (5' --> 3')
Actb	CTT CCT TCC TGG GTA TGG AAT C	CTG TGT TGG CAT AGA GGT CTT
Chrm2	CAA GTC TGG GCC TAA TGG ATA G	GAA TGG CAG AGA GGG AAT CAA
Slc2a4	ACT TAG GGC CAG ATG AGA ATG	GTA AGG GAA GAG AGG GCT AAA G
Mmp12	CTG GTT CGG TTG TTA GGA AGA	CCC TGA GCA TAC AGT GGA TAT G
Gja1	CCT CAC CAA ACG GCT TCT ATC	TCA CCT CCC TGA TGC TAA CT
Sod2	TAA GGA TGG ATG GAG TGG TAG A	CCT AGC TTG GGT CTG TTG ATT
Ndufs2	CGC CTT TCT TCT GGA TGT TTG	ACT CCT CCT GGT CGG ATA TAA

Supp fig 3. Rat primer sequences used for qPCR of select DEGs.

Supplemental figure 4

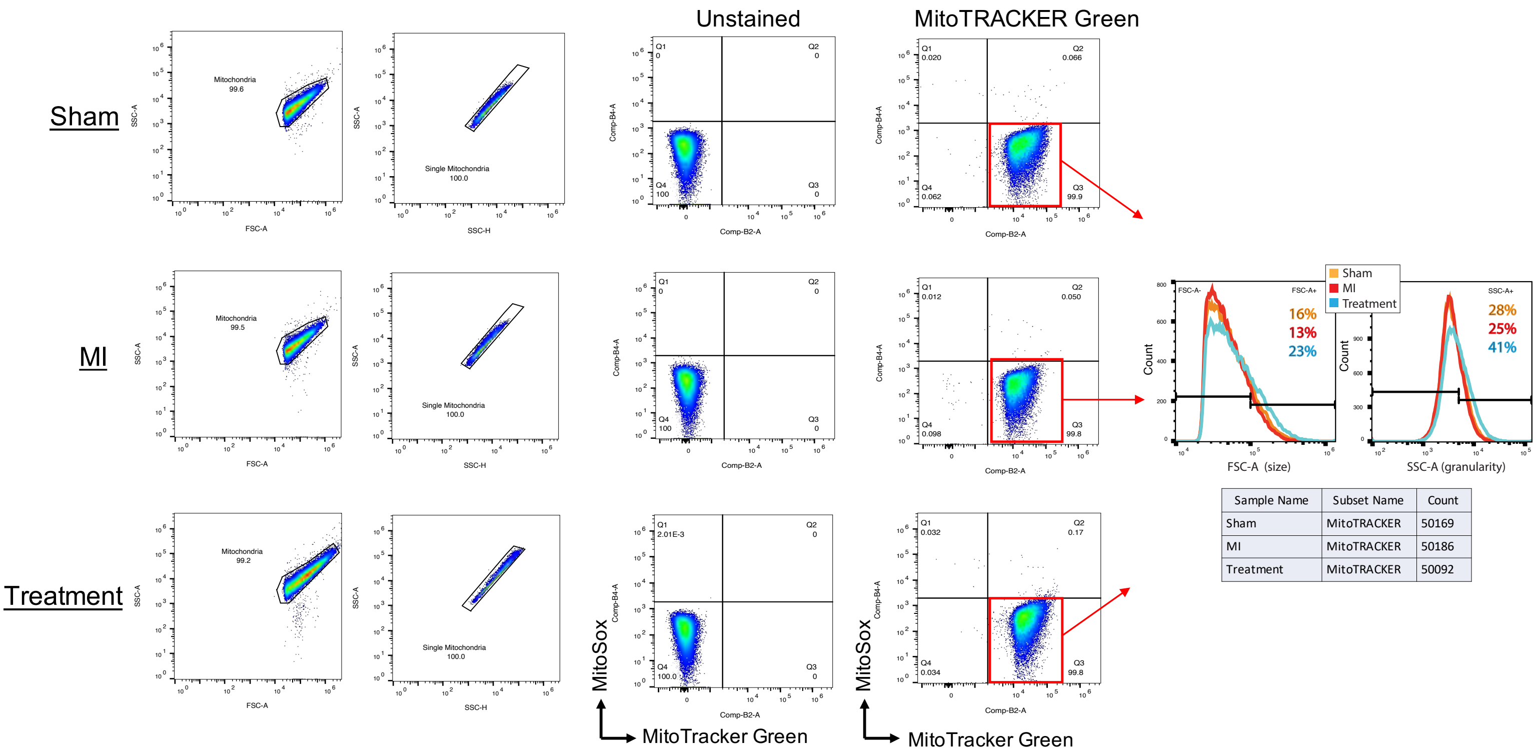

Sample Name	Subset Name	Count
Sham	MitoTRACKER	50169
MI	MitoTRACKER	50186
Treatment	MitoTRACKER	50092

Supp fig 4. Representative gating strategy for flow cytometric mitochondrial forward and side scatter analysis depicting size and granularity, respectively. 50,000 +/- MitoTracker Green positve mitochondria per animal were included in distribution analysis.

Supplemental figure 5

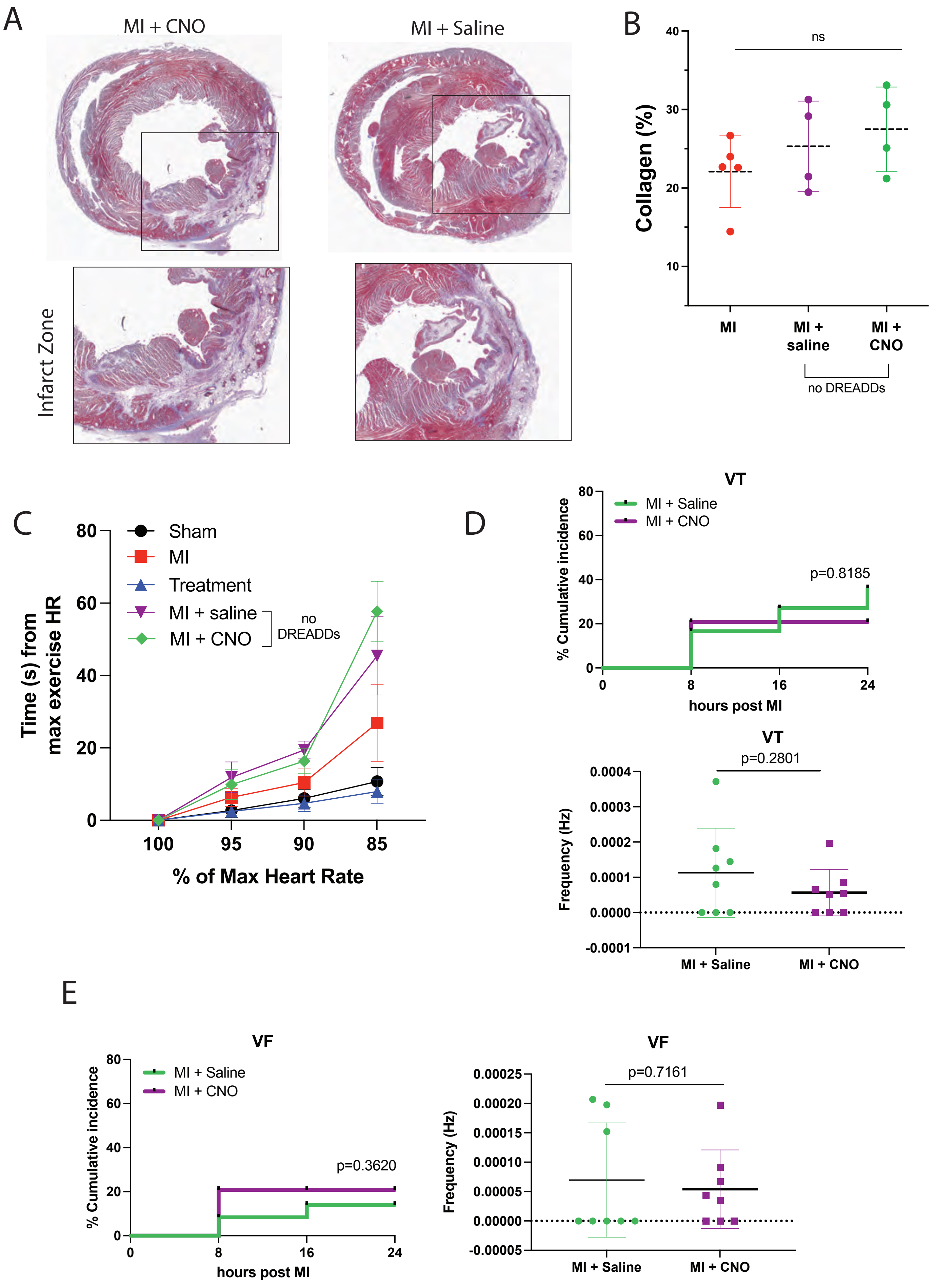

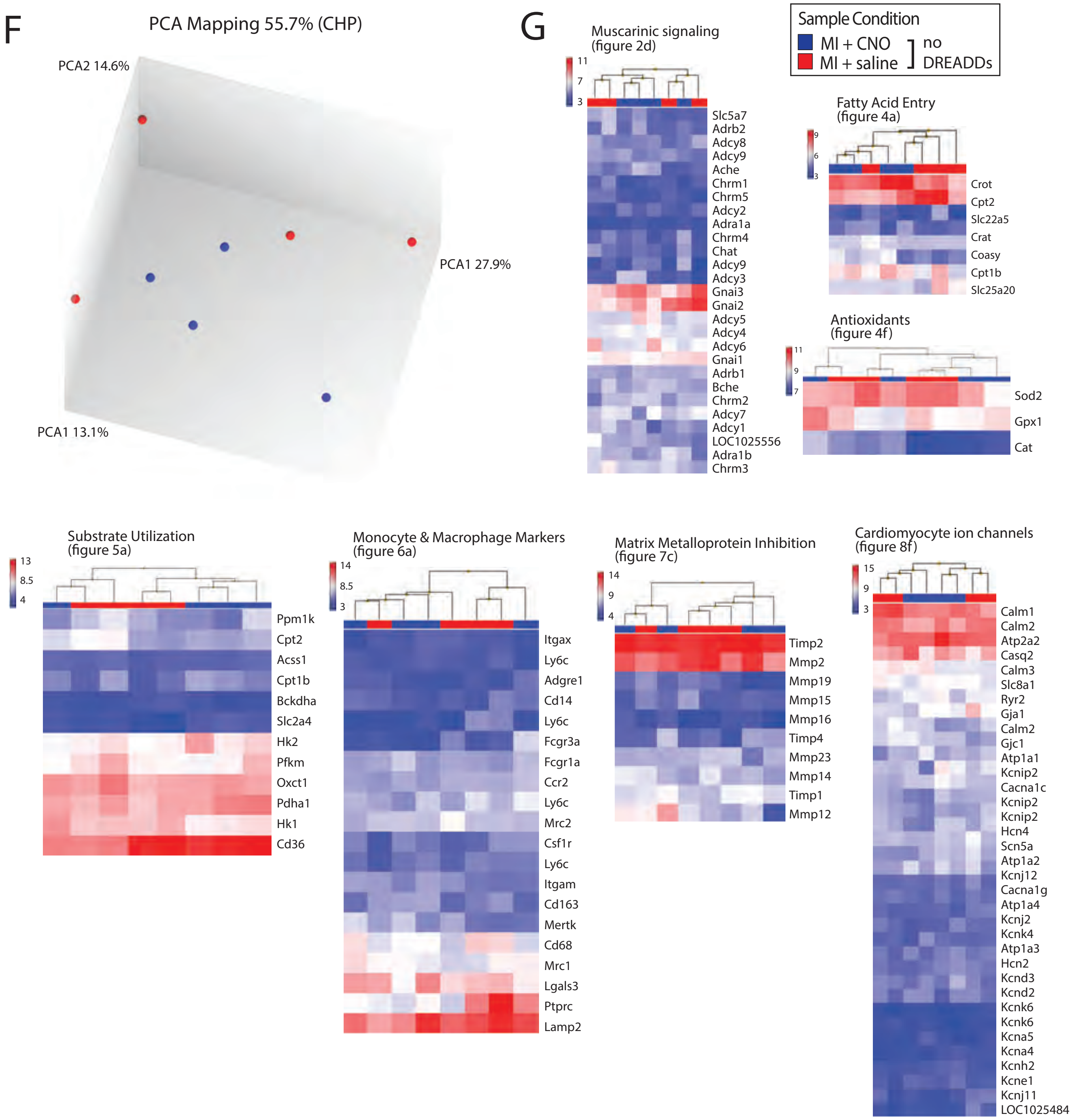

Supp fig 5. Select outcomes of additional control MI groups not expressing DREADDs and receiving either saline or CNO injections. **(A-B)** Representative Masson's trichrome images of myocardial sections from MI+saline and MI+CNO animals are shown in each column. Large and small bounding boxes on images of the full section (top row) indicate the bounding area of the high-resolution images for the infarct zone (bottom row). Blue denotes the presence of collagen. Percent collagen content **(B)** within a region of the infarct zone was measured using the trichrome images (MI + saline, n=4; MI + CNO, n=4; one-way ANOVA; mean±SD; *p<0.05). **(C)** HRR time 5 days after MI as a percentage of HR at peak running effort (the maximum HR). Recovery time to 95%, 90%, and 85% of maximum HR was significantly longer for all 3 groups of MI animals compared to Sham or Treatment groups. HRR time was not significantly different between Treatment and Sham animals (Sham n=24; MI, n=9; Treatment, n=13; MI+saline, n=8; MI+CNO, n=8. **(D-E)** Cumulative incidence and frequency of **(D)** Ventricular Tachycardia (VT) and **(E)** Ventricular Fibrillation are not different between MI groups with or without DREADDs (n=8 each group). **(F)** Transcriptome PCA of MI animals not expressing DREADDs (MI+saline, n=4; MI+CNO, n=4). **(G)** Transcriptome expression heatmaps and hierarchical clustering of key pathways and genes involved in muscarinic signaling (main text figure 2d), mitochondrial respiration (main text figure 4a), antioxidants (main text figure 4f), substrate utilization (main text figure 5a), inflammation (main text figure 6a), ECM remodeling (main text figure 7c), and cardiomyocyte ion channels (main text figure 8f).

Supplemental figure 6

GAPDH (bottom portion of cut membrane includes 70 - 8 kDa; stripped after probing for Il-1b)

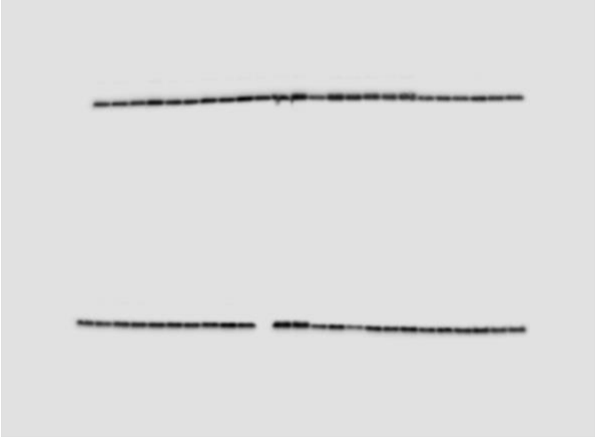

Il-1b (bottom portion of cut membrane includes 70 - 8 kDa)

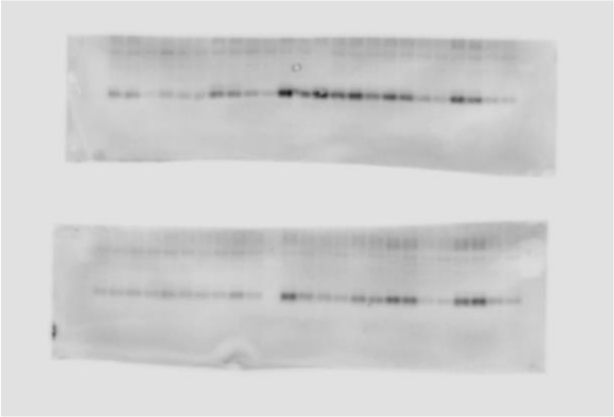

Serca2a (top portion of cut membrane includes 260 -70 kDa)

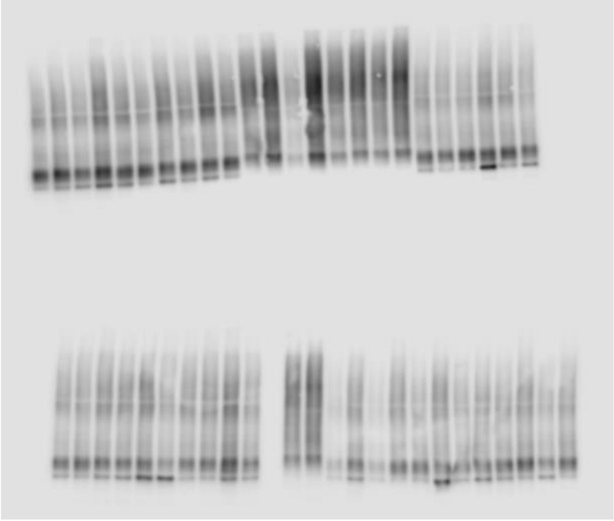

Supplement: Supp Mat #1 [file NIHMS1936596-supplement-Supp_Mat__1.pdf]
